# One hundred years of Ecuadorian biomedical scientific output and its association with the main causes of mortality: a bibliometric study

**DOI:** 10.3389/fmed.2024.1395433

**Published:** 2024-07-03

**Authors:** Ivan Sisa, Jhon Caicedo-Potosí, María Cordovez, Clara Verdezoto, Mishell Barreno, Martín Coral, Gricelda Herrera-Franco

**Affiliations:** ^1^Colegio de Ciencias de la Salud, Escuela de Medicina, Universidad San Francisco de Quito USFQ, Quito, Ecuador; ^2^Centro de Investigación y Proyectos Aplicados a las Ciencias de la Tierra (CIPAT), Escuela Superior Politécnica del Litoral (ESPOL), Guayaquil, Ecuador; ^3^Facultad de Ciencias de la Ingeniería, Universidad Estatal Península de Santa Elena, La Libertad, Ecuador

**Keywords:** scientific production, health research system, bibliometric, Ecuador, burden of disease

## Abstract

**Introduction:**

Historically, low-and middle-income countries have been scarce producers of biomedical research; only 2% of the global scientific output is produced by these countries despite accounting for 92% of the global burden of disease. In addition, few low-and middle-income countries have exhaustively mapped and analyzed their scientific production in health and its association with main local burden of disease.

**Objective:**

To evaluate the evolution of biomedical research in Ecuador over the last 100 years and its relationship with the main causes of mortality.

**Methods:**

A bibliometric study embedded in a systematic review design was carried out using biomedical publications indexed in Scopus and Web of Science (WoS) during the period 1920–2021. Information from the National Institute of Statistics and Census was used to identify the main causes of mortality.

**Results:**

Our search strategy identified 16,697 publications related to biomedicine in Ecuador. Of these 3,225 articles met the criteria for this study. Since 2010, there has been an exponential increase in scientific production in biomedicine. This increase was predominantly based on cross-sectional observational studies (49.67%). During the period analyzed (1920–2021), biomedical production was distributed with 52.43% in clinical research, 37.79% in public health, and 9.77% in basic sciences. The research focus with the highest number of publications was epidemiology and surveillance system of diseases (23.44%). Additionally, private universities are the largest producers of biomedical research compared to public universities, 40.12% vs. 19.60%, respectively. Of the total biomedical research produced, 18.54% is associated with the main causes of mortality, and the Ecuadorian private university is the largest contributor to these studies compared to public universities, 39.97% vs. 16.72%.

**Conclusion:**

In one century, Ecuador produced 3,225 articles in biomedicine, according to our criteria. 18.54% of the total produced is aimed at solving the main causes of mortality in the country. Private universities are the leaders in scientific production related to health in Ecuador.

## 1 Introduction

An appropriate and efficient national health research system should address both the health problems of importance to its population and the interventions and outcomes considered important by patients and clinicians (1, 2). However, in reality, there is a tremendous mismatch between the actual health needs and the research conducted, which has been described as one of the factors contributing to the estimated 85% waste in biomedical research globally (2). This waste in futile investigations is equivalent to $200 billion of the approximately $240 billion invested in health research and development (R&D) in 2010 (2, 3). For example, in the last century cardiovascular disease (e.g., ischemic heart disease and stroke) has become the leading cause of premature mortality and morbidity globally (4), and low-and middle-income countries (LMICs) are estimated to contribute to ~80% of the global cardiovascular disease burden (5). However, LMICs contributed to only 0.2% of total cardiovascular disease publications, leaving their populations without easily accessible evidence to make informed clinical decisions about their own health (2, 6). Historically, LMICs have been scarce producers of biomedical research; they produce only 2% of global scientific output (7). For example, Perel et al. found a poor correlation between burden of disease and research output produced as Randomized Clinical Trials (RCTs) in Latin America (8). Ecuador, considered by the World Bank as a middle-income country in Latin America, is no exception. Despite being a middle-income country, it struggles with diseases characteristic of low-income countries, such as chronic malnutrition and lower respiratory infections (9). Besides, according to the Ecuadorian National Institute of Statistics and Census (INEC), in 2021 the main causes of death were COVID-19-related diseases, ischemic heart disease, and diabetes mellitus (10). During the last 15 years, Ecuador has undergone important reforms in the areas of health and higher education. In terms of health, a new model (MAIS-FCI, Spanish acronym) of care focused on primary health care was implemented. This model, as well as the National Health Research Policy issued in 2020 by the Ecuadorian Ministry of Public Health (MSP), establishes that all research efforts in the country should be conducted to provide solutions to its main health problems (11). In terms of higher education, the Organic Law of Higher Education (LOES) was issued in 2010, which sought to strengthen the research capacity of Ecuadorian universities through multiple regulations and programs. Likewise, the LOES promotes that all research and teaching efforts carried out at the local level should be aligned with the main health, economic, and social needs of Ecuador (12, 13). Academia is the driving force behind research and is naturally called upon to produce knowledge that responds to the particular needs of society (14). However, there is little evidence of how academia has contributed to solving the main causes of morbidity and mortality in Ecuador. According to the literature, only two bibliometric analyses have addressed this issue, and their main findings are that during the last decade, scientific production related to health sciences in Ecuador showed an exponential growth. However, this increase has been at the expense of a decrease in the quality of the evidence generated through cohort and randomized clinical trial studies, and with no relation to the main causes of mortality in the Ecuadorian population (15, 16). The weaknesses of these previous studies are the short period of time analyzed and the possibility of missed publications due to the use of a single database (Scopus). The present study seeks to answer the following research questions:

- How has biomedical research evolved in Ecuador during the last century?
- Are Ecuadorian universities contributing to the solution to the main causes of mortality in the country?

A systematic literature review design embedded in a bibliometric analysis study was used to answer these questions.

## 2 Methods

A bibliometric study embedded in a systematic review design was carried out in three phases: (i) extraction/identification of articles in the Scopus and Web of Science (WoS) databases, (ii) construction of the database and extraction of previously defined variables, and (iii) statistical analysis and generation of results.

### 2.1 Phase I: information extraction/identification

**Figure 1:**
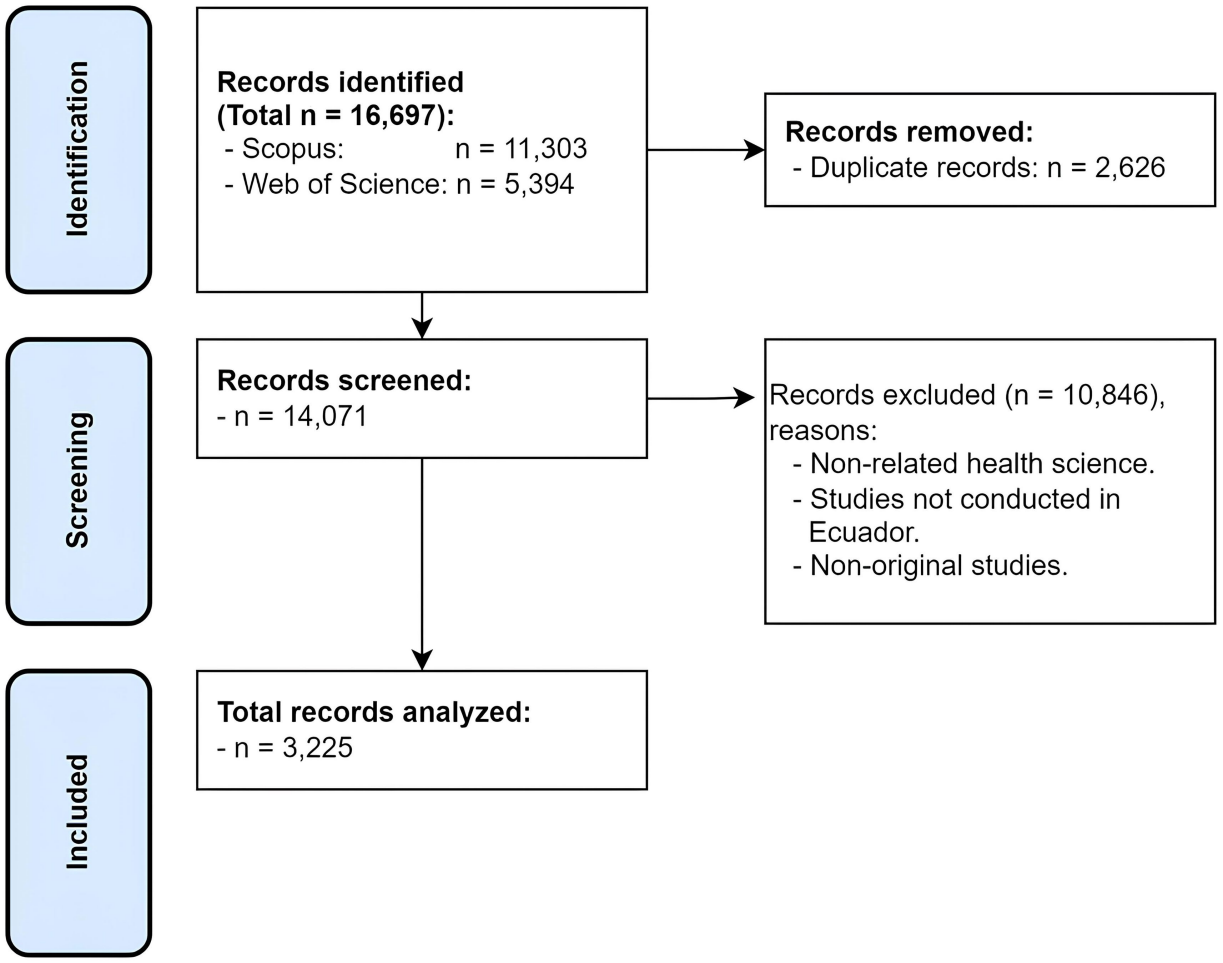
PRISMA flowchart.

In the Scopus and WoS databases, a search equation was applied to identify the largest possible number of articles related to biomedical research produced in Ecuador between 1920 and 2021. Scopus and WoS were selected because of their high quality and coverage of scientific journals. Additionally, WoS is the oldest research and citation database in the world and has the capability to export search results (17, 18). The search equations are available in the Supplementary material accompanying this publication (Supplementary Table S1). Initially, 11,303 publications were identified in Scopus and 5,394 publications in WoS. The Bibliometrix tool RStudio (19) was used to combine both databases and eliminate duplicate documents (*n* = 2,626), generating a total of 14,071 articles (Figure 1).

### 2.2 Phase II: construction of the database and extraction of variables

During this phase of the study, five members of the research team performed the manual screening of the studies identified in the first phase (*n* = 14,071). The following inclusion criteria were applied: (i) studies related to health sciences and with application in humans, (ii) studies carried out by an Ecuadorian institution, and (iii) studies that used data from Ecuador in their analysis. On the other hand, the following exclusion criteria were applied: (i) articles not available in their full version by institutional/library access or on the web as open access and (ii) non-original studies (e.g., letter to the editor, commentary, poster). Thus, the number of studies eligible for the final analysis was 3,225 documents. Additionally, a disambiguation process was carried out to unify the affiliations of the Ecuadorian universities that registered publications in biomedical sciences as described in the literature (20). For the extraction of the variables, an electronic form was developed and tested in Microsoft Excel. The following variables were recorded in this form: (i) research focus (basic science, clinical, and public health), (ii) study design (case or series report, case–control study, cohort study, cross-sectional/survey study, ecological study, meta-analysis/network meta-analysis, randomized clinical trial study, and other, including laboratory studies, basic science, and modeling, (iii) quartile placement according to SCImago Journal Rank (SJR), as a proxy measure of impact and quality (21), (iv) institutional affiliation and type of funding (public, private with state allocation, and private self-financed), (v) line of research, (vi) journal of publication and the number of discontinued journals in Scopus, (vii) geographic location by province, (viii) international collaboration, and (ix) relation with the main causes of mortality in the country. To identify the main causes of mortality, information from the annual reports of the Ecuadorian National Institute of Statistics and Censuses (INEC) was used, and 5 standardized rankings of causes of mortality per decade were constructed. Finally, to assure consistency and accuracy of extracted data, the last author proofread randomly chosen extracted data. Discrepancies in data screening were discussed and resolved by consensus between the first and last authors.

### 2.3 Phase III: statistical analysis and generation of results

Descriptive statistics were used to summarize the characteristics of the articles analyzed. To explore trends over time in scientific production per institution and mortality, the entire period analyzed was divided into four periods of 25 years each and five periods of approximately 10 years each (with the exception of 1920–1979), respectively. The chi-square test was used to assess differences between categorical variables. To compare university-specific research production, a standardized rate of the number of publications per 1,000 students was calculated. The number of health-related students per university was obtained from the Sistema Integral de Información de la Educación Superior.^1^ The statistical program used for data processing and analysis was RStudio for Windows, V.4.2.2. To claim statistical significance, we used a *p*-value of < 0.05. In addition, ArcGIS Pro V.3.1.2 was used to map the international collaboration of the publications and their geographic distribution at the local level by province.

## 3 Results

The search strategy used identified 16,697 publications (11,303 from Scopus and 5,394 from WoS) related to biomedicine in Ecuador. A total of 13,472 articles were excluded (duplicate articles *n* = 2,626, and articles that did not meet the inclusion criteria *n* = 10,846), so the final sample analyzed was 3,225 articles related to biomedicine (Figure 1).

### 3.1 Biomedical production trend over time

**Figure 2:**
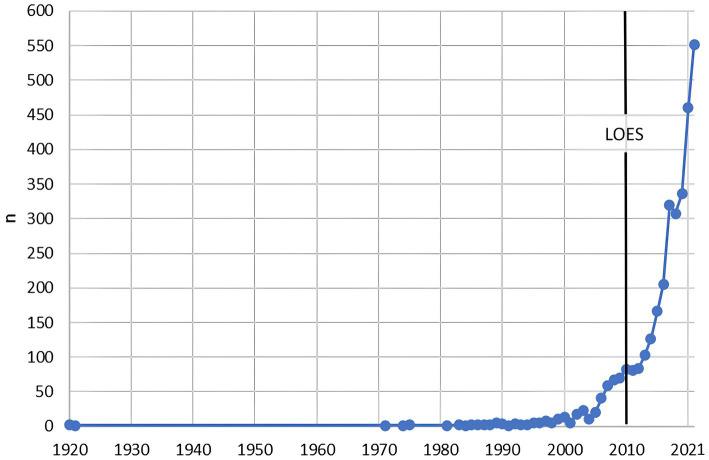
Ecuadorian trend of biomedical publications from 1920 to 2021. The solid vertical line shows the year of the launching of the Higher Education Law (LOES, Spanish acronym).

**Figure 3:**
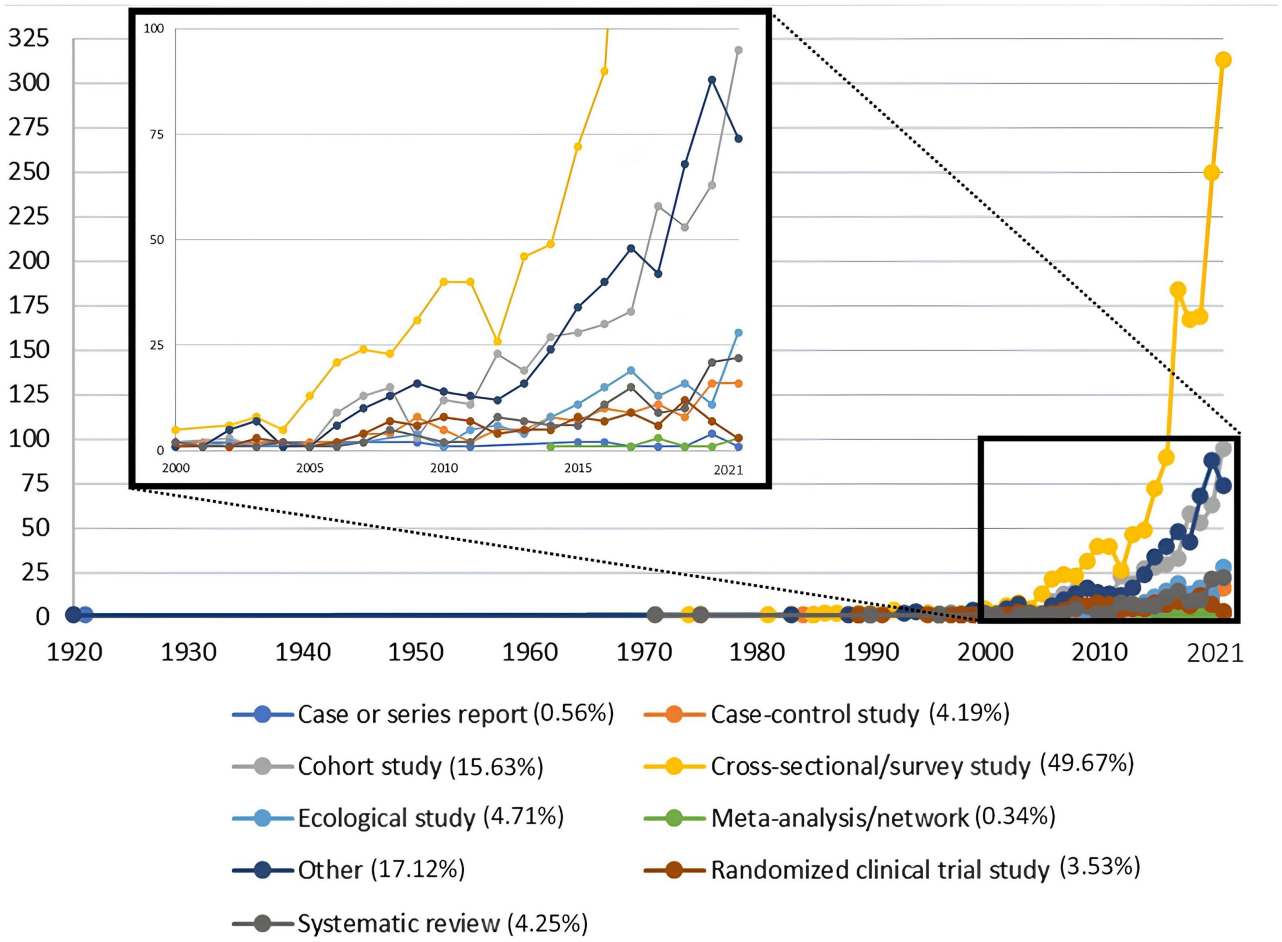
Trend of biomedical production in Ecuador according to the study design used (*n* = 3,225). The published studies that did not use a classic epidemiological design were counted in the “other” category; this category includes mainly basic science studies conducted in a laboratory setting.

According to the period analyzed, there was a linear trend of low production until the beginning of the 2000s and an exponential increase at the end of the same decade (Figure 2). The most frequently used type of epidemiological design was cross-sectional (49.67%), followed by other (17.11%) and cohort studies (15.63%). There was a downward trend in the production of randomized clinical trial studies (Figure 3). Additionally, the research area with the highest production was related to clinical areas (52.43%, *n* = 1,691/3,225) (Table 1). When analyzing the scientific production by time periods, we found that during periods I and II (1920–1969), there was a scarce production of articles (0.09%, *n* = 3), mainly in the areas of intestinal parasitosis and tropical diseases such as yellow fever. During period III (1970–1994), the production increased to 1.08% (*n* = 35) of the total analyzed. In this period, scientific production stood out mainly in the clinical areas (45.71%, *n* = 16). Also in this period, the first randomized clinical trial conducted in Ecuador (published in 1989 in the British Journal of Obstetrics and Gynaecology) was recorded. This study assessed whether calcium supplementation reduced the occurrence of pregnancy-induced hypertension. In period IV (1995–2021), an exponential production of biomedical research in the country was evidenced, concentrating 98.82% (*n* = 3,187) of all that was produced in the last century. In this period, research in the clinical area continued to dominate, with 52.49% (*n* = 1,673) of the total produced; however, it is worth mentioning that the production of research at the basic biomedical sciences level was 9.63% (*n* = 307) (Supplementary Tables S2–S5). Overall, the scientific production of the analyzed period was encompassed in 1,157 scientific journals. Currently, 6.57% (76/1,157) of those journals are discontinued in the Scopus database (Supplementary Table S6). The scientific journals with the highest number of Ecuadorian publications related to biomedicine in descending order are: Archivos Venezolanos de Farmacología y Terapéutica (2.76%, *n* = 89), Revista Ecuatoriana de Neurología (2.36%, *n* = 76), and American Journal of Tropical Medicine and Hygiene (2.01%, *n* = 65) (Supplementary Table S7).

**Table 1:** Study characteristics and main producers of Ecuadorian biomedical sciences-related publications, 1920–2021.

Characteristic	Total *n* = 3,225	Public University *n* = 632	Private University *n* = 1,294	Mixed University *n* = 331	Non-academic Institution^¶^ *n* = 968	*p*-value^*^
SCImago journal rank, *n* (%)						<0.001
Q1	1,397 (43.32)	239 (37.81)	538 (41.57)	119 (35.95)	501 (51.75)	
Q2	738 (22.88)	134 (21.20)	296 (22.87)	79 (23.87)	229 (23.65)	
Q3	421 (13.05)	90 (14.24)	174 (13.45)	37 (11.18)	120 (12.40)	
Q4	579 (17.95)	152 (24.05)	238 (18.39)	87 (26.28)	102 (10.54)	
None	90 (2.79)	17 (2.69)	48 (3.71)	9 (2.72)	16 (1.65)	
Study design, *n* (%)						–
Case/series report	18 (0.56)	3 (0.47)	6 (0.46)	3 (0.91)	6 (0.62)	
Ecological	152 (4.71)	20 (3.16)	23 (1.78)	9 (2.72)	100 (10.33)	
Cross-sectional/survey	1,602 (49.67)	331 (52.37)	676 (52.24)	187 (56.50)	408 (42.15)	
Case-control	135 (4.19)	23 (3.64)	80 (6.18)	19 (5.74)	13 (1.34)	
Cohort	504 (15.63)	51 (8.07)	253 (19.55)	36 (10.88)	164 (16.94)	
Randomized clinical trial	114 (3.53)	35 (5.54)	27 (2.09)	3 (0.91)	49 (5.06)	
Systematic review	137 (4.25)	24 (3.79)	40 (3.09)	9 (2.72)	64 (6.61)	
Meta-analysis	11 (0.34)	1 (0.15)	2 (0.15)	–	8 (0.82)	
Other^¶¶^	552 (17.12)	144 (22.78)	187 (14.45)	65 (19.64)	156 (16.11)	
Research focus, *n* (%)						<0.001
Basic science	315 (9.77)	71 (11.23)	140 (10.82)	38 (11.48)	66 (6.82)	
Clinical	1,691 (52.43)	297 (46.99)	719 (55.56)	171 (51.66)	504 (52.07)	
Public health	1,219 (37.79)	264 (41.77)	435 (33.62)	122 (36.86)	398 (41.12)	

^*^Categorical data was analyzed using chi-square test.

^¶^Non-academic institutions encompass hospitals/clinics, governmental agencies, and NGOs.

^¶¶^This category refers to research conducted in a laboratory setting (traditional medicine, genetic/molecular analysis) or any kind of computational modeling research.

### 3.2 Main producers of biomedical research

**Figure 4:**
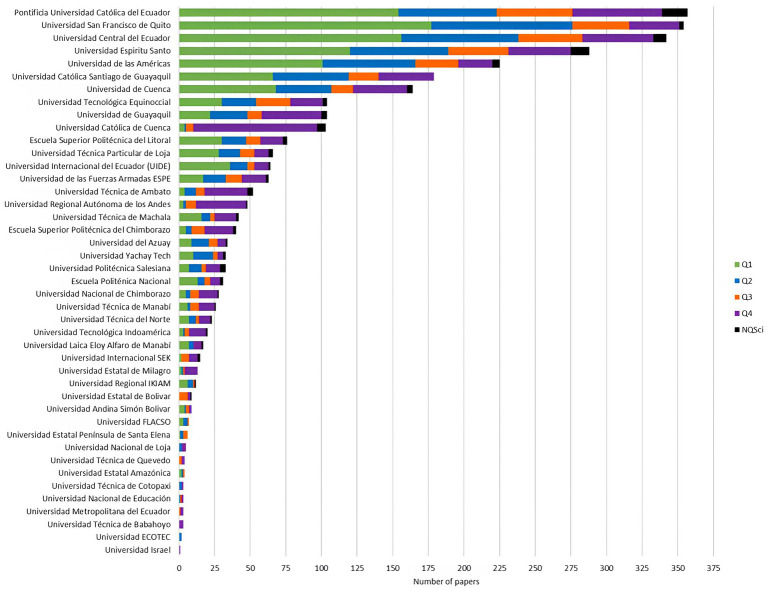
Distribution of Ecuadorian biomedical publications by university and SCImago ranking quartile.

**Figure 5:**
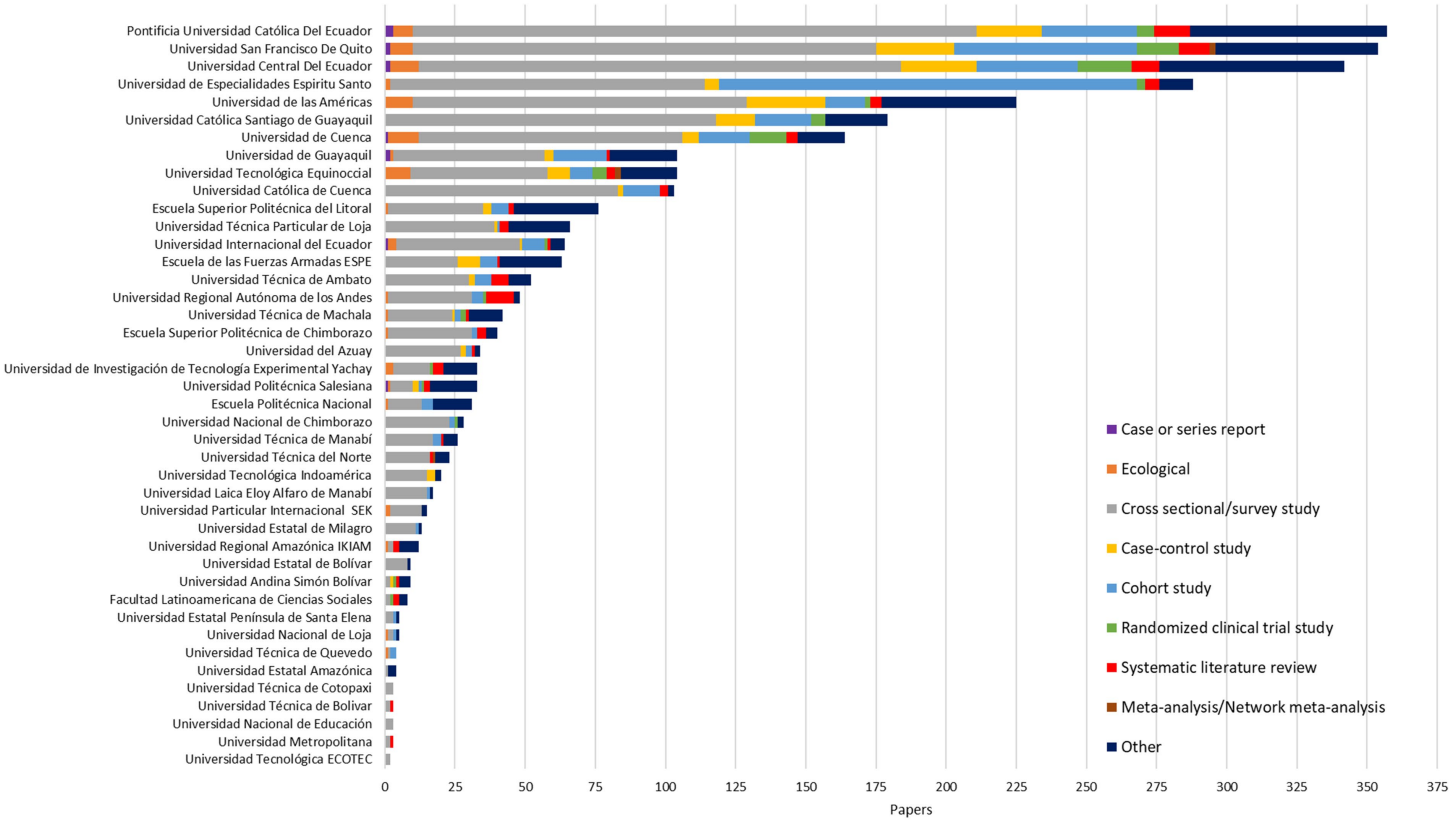
Distribution of Ecuadorian biomedical publications by type of design study used and university.

Overall (1920–2021), private universities were the largest producer of biomedical research at the local level, followed by non-academic institutions (hospitals/clinics, ministry of public health, and non-governmental organizations), 40.12% (*n* = 1,294) vs. 30.01% (*n* = 968), respectively (Table 1). Regarding the impact and quality of publications, non-academic institutions had the highest percentage of publications in Q1 journals, followed by private universities, 51.75% vs. 41.57%, respectively; *p*-value < 0.001 (Table 1). When analyzing the data by Higher Education Institutions (HEIs) only, we found that the three universities with the highest production were: Pontificia Universidad Católica del Ecuador (11.06%, *n* = 357), Universidad San Francisco de Quito USFQ (10.97%, *n* = 354), and Universidad Central del Ecuador (10.60%, *n* = 342) (Figure 4). This trend varied after standardizing research output by number of health-related students; thus, Universidad San Francisco de Quito USFQ and Universidad Espíritu Santo had the highest production per 1,000 students—295 and 239 publications, respectively (Supplementary Table S8). It is worth mentioning that Pontificia Universidad Católica del Ecuador, Universidad San Francisco de Quito USFQ, and Universidad Central del Ecuador have undergraduate and graduate programs in health sciences, with Universidad Central del Ecuador in operation for more than 180 years (Supplementary Table S8). However, other higher education institutions without a faculty or academic college for the study of medicine/health sciences also registered biomedical publications, as was the case of the Escuela Superior Politécnica del Litoral (ESPOL) (Figure 4). The cross-sectional design was the most used by both researchers and universities, with a predominance of research carried out in collaboration between private and public universities (56.50%) (Mixed University) (Table 1; Figure 5). Studies with greater methodological complexity, such as cohort studies and randomized clinical trials, were carried out in a higher percentage by private and public universities, 19.55% vs. 5.54%, respectively. It is worth mentioning that public universities in the country registered a higher percentage of research classified as “other” (22.78%, *n* = 144). This category encompasses research conducted in a laboratory facility to study traditional medicine, genetic/molecular analysis, or studies of any kind of computational modeling research. This finding was aligned with the data by research focus, where public universities and mixed collaboration (public and private) had a high percentage of publications in basic sciences (22.71%). In contrast, private universities had a higher contribution in the area of clinical sciences, with 55.56%. Finally, public universities together with non-academic institutions registered the highest contributions in the area of public health at the local level, 41.77% vs. 41.12%, respectively (Table 1).

### 3.3 Lines of research and focus on the main causes of mortality

**Figure 6:**
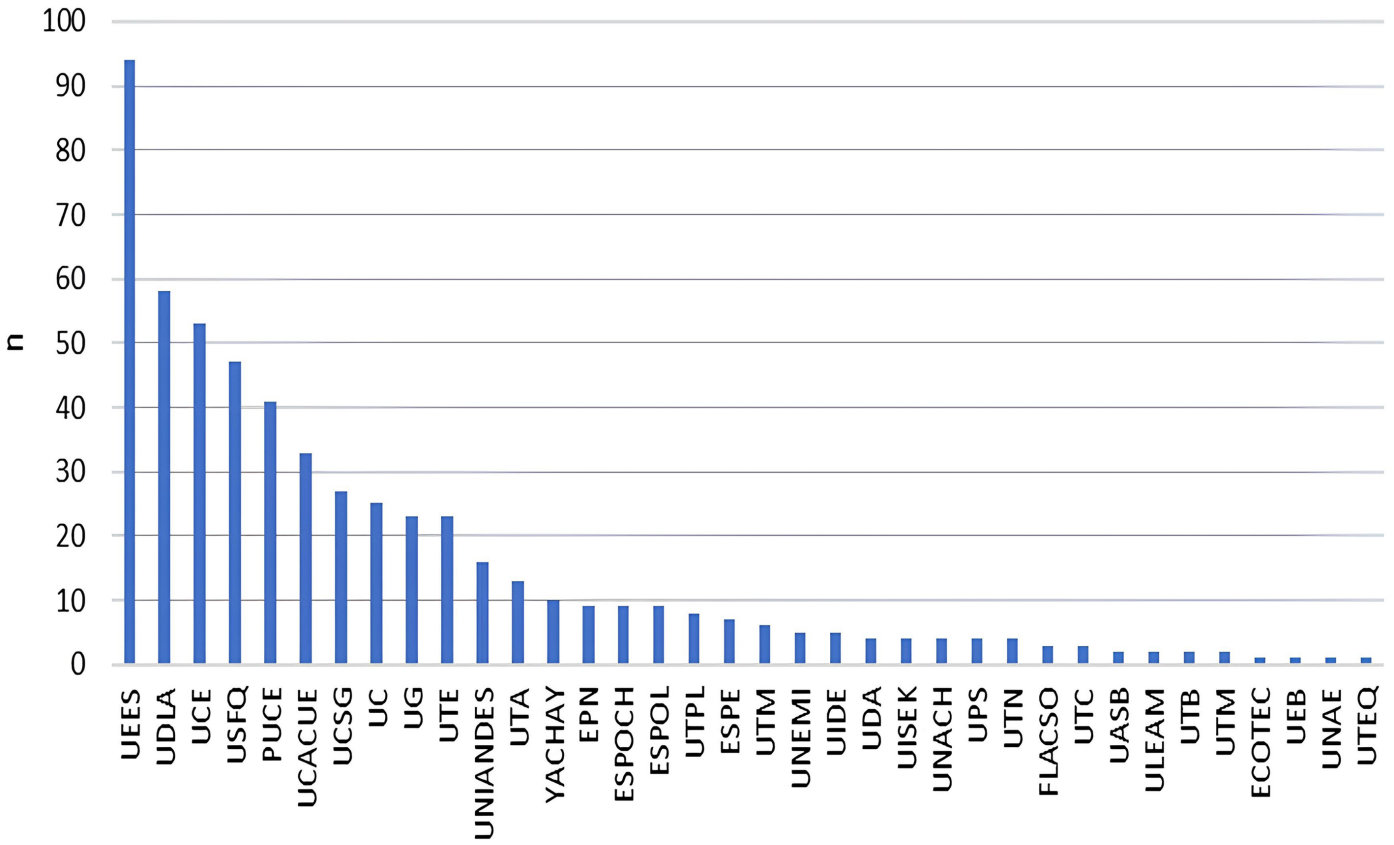
Distribution of biomedical production related to the main causes of mortality by higher education institutions.

**Figure 7:**
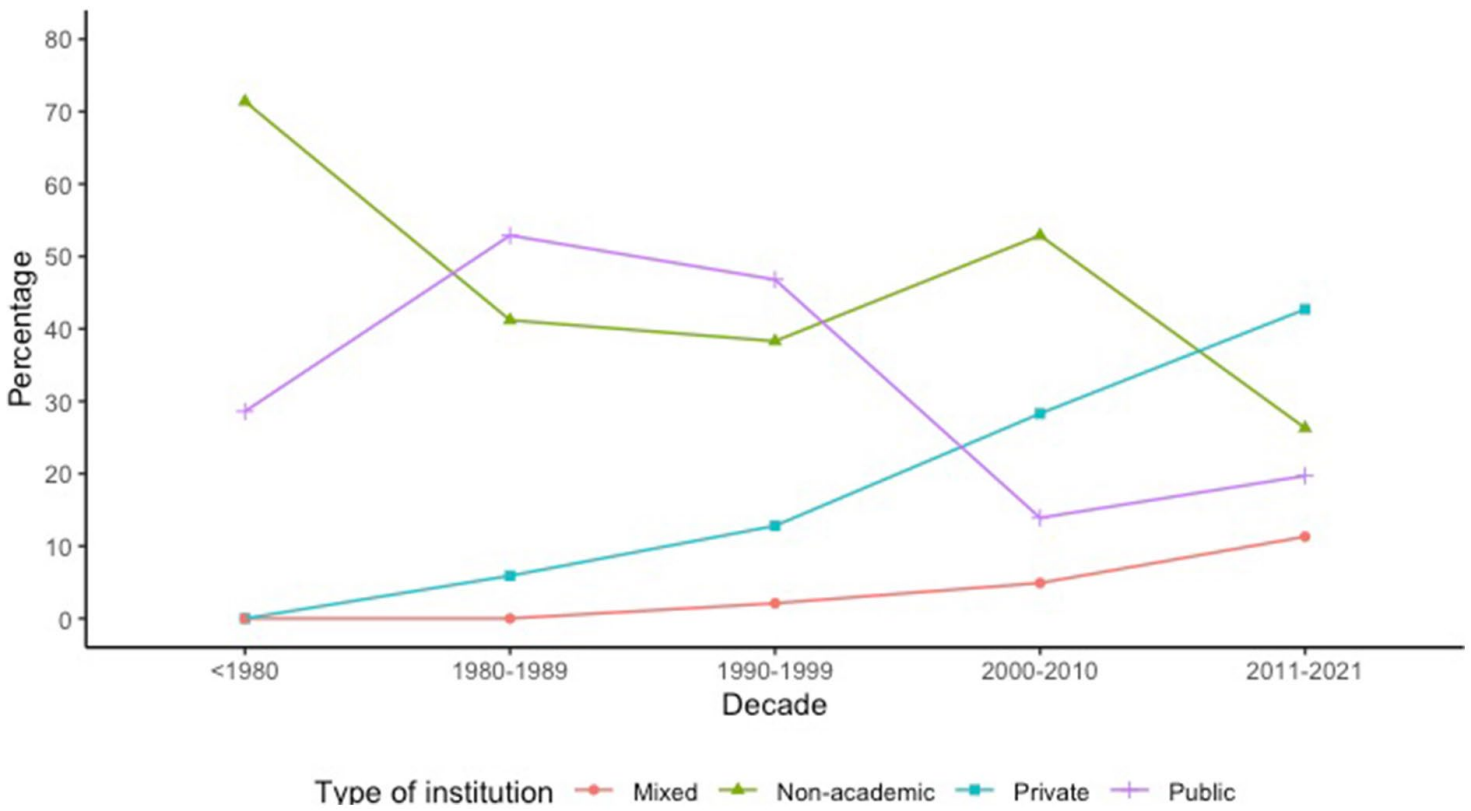
Time trend of Ecuadorian biomedical publications by institution producer (*n* = 3,225).

The analysis of the lines of research shows that 23.44% of the publications focus on epidemiology and surveillance system of diseases, followed by diagnosis and treatment research (17.21%) and research on causes of diseases (16.62%) (Table 2). Public universities in Ecuador, compared to private universities and non-academic institutions, publish more in the lines of research related to early detection and prevention research (5.85%), psychosocial aspects of diseases (15.66%), and other (4.43%). On the other hand, private universities focus on research related to research on causes of diseases (21.33%), and non-academic institutions focus mainly on epidemiology and surveillance system of diseases (26.55%) (Table 2). According to INEC, 18.54% (*n* = 598) of the total research produced during the century 1920–2021 was associated with the main causes of mortality. Sixty-two percent of these publications had clinical areas as their main research focus (Supplementary Figure S1). The private university (self-and co-financed) in Ecuador contributed the most to this research compared to public universities, 39.97% (*n* = 239/598) vs. 16.72% (*n* = 100/598), respectively. For example, the three universities with the highest contributions were Universidad Espíritu Santo (UEES), Universidad de las Américas (UDLA), and Universidad Central del Ecuador (UCE) (Figure 6). When removing the publications related to COVID-19 (*n* = 194), the trend of the four largest producers of studies related to the main causes of mortality maintained their trend, two private universities with one public university (UESS, UCE, and USFQ in order of frequency) as the largest producers of studies related to the main causes of mortality was maintained (Supplementary Figure S2). Thus, only 15.59% (63/404) of public university production is on track to resolve main local causes of mortality compared to private universities at 38.61% (156/404) (Supplementary Figure S3). Table 3 shows the percentage of biomedical research output dedicated to addressing the leading causes of mortality per decade in Ecuador. Hence, before the 80s, 42.85% of research production was aligned with the main burden of disease for that period of time, and the main producer was non-academic institutions (*n* = 5) like International Health Board. Meanwhile, the lowest research output (10.73%) was seen at the beginning of the year 2000, and the main producers during this period were non-academic institutions (*n* = 217) and private universities (*n* = 116) (Supplementary Table S9). It is worth mentioning that during the years 2011–2021, the scientific production related to the main and historical causes of mortality (e.g., cardiovascular disease, diabetes, cancer, etc.) was over-represented due to the COVID-19 effect. Thus, after removing COVID-19 related publications, the percentage of scientific output dedicated to addressing classical main causes of mortality during 2011–2021 decreased to 12.43%. The following causes of mortality were the highest researched per decade: infectious and parasitic disease (*n* = 3, 42.85% [years <1980]), other protein-energy malnutrition (*n* = 3, 17.6% [years 1980–1989]), malnutrition (*n* = 6, 12.8% [years 1990–1999]), certain conditions originating in the prenatal and perinatal period (*n* = 10, 2.4% [years 2000–2010]), and diabetes mellitus (*n* = 59, 2.1% [years 2011–2021]). Regarding which institutions were the main scientific drivers of publications aligned with the principal causes of mortality, we found that non-academic and public institutions led the production at the beginning of the analysis period. However, after the decade 2000, private universities took the lead as the main scientific producers in the country, and this trend is consistent after analyzing overall production and only INEC-related publications (Figure 7; Supplementary Figure S4).

**Table 2:** Distribution of Ecuadorian biomedical sciences-related publications by research line and type of university.

Research lines, *n* (%)	Total *n* = 3,225	Public University *n* = 632	Private University *n* = 1,294	Mixed University *n* = 331	Non-academic Institution *n* = 968
Epidemiology and surveillance system of diseases	756 (23.44)	135 (21.36)	268 (20.71)	96 (29.00)	257 (26.55)
Research on causes of diseases	536 (16.62)	93 (14.71)	276 (21.33)	54 (16.31)	113 (11.67)
Biology research of diseases (molecular and cellular mechanisms)	387 (12)	81 (12.81)	173 (13.37)	45 (13.60)	88 (9.09)
Early detection and prevention research	176 (5.46)	37 (5.85)	69 (5.33)	18 (5.44)	52 (5.37)
Diagnosis and treatment research	555 (17.21)	99 (15.66)	178 (13.76)	47 (14.20)	231 (23.86)
Provision of health services	177 (5.48)	36 (5.70)	57 (4.40)	12 (3.63)	72 (7.44)
Psychosocial aspects of diseases	384 (11.90)	99 (15.66)	194 (14.99)	43 (12.99)	48 (4.96)
Public policy	151 (4.68)	24 (3.80)	44 (3.40)	8 (2.42)	75 (7.75)
Other	103 (3.19)	28 (4.43)	35 (2.70)	8 (2.42)	32 (3.31)

**Table 3:** Distribution of Ecuadorian biomedical research output and leading causes of mortality per decade, 1920–2021.

Ranking	<1980 *n* = 7	*n* (%)	1980–1989 *n* = 17	*n* (%)	1990–1999 *n* = 47	*n* (%)	2000–2010 *n* = 410	*n* (%)	2011–2021 *n* = 2,744	*n* (%)
1	Infectious and parasitic diseases	3 (42.85)	Intestinal infectious diseases	1 (5.9)	Cerebrovascular disease	2 (4.3)	Cerebrovascular disease	3 (0.73)	Diabetes mellitus	59 (2.1)
2	Tuberculosis	–	Pneumonia	–	Pneumonia	1 (2.1)	Diabetes mellitus	5 (1.21)	Hypertension	48 (1.7)
3	Intestinal infectious diseases	–	Cerebrovascular disease	–	Intestinal infectious diseases	2 (4.3)	Hypertension	5 (1.21)	Cerebrovascular disease	54 (1.4)
4	Heart diseases	–	Bronchitis, emphysema, and asthma	–	Ischemic heart disease	–	Influenza and pneumonia	9 (2.2)	Dementia and Alzheimer’s disease	9 (0.32)
5	Cancer	–	Road traffic injuries	–	Road traffic injuries	–	Ischemic heart disease	1 (0.24)	Road traffic injuries	16 (0.5)
6	Vascular diseases of the central nervous system	–	Ischemic heart disease	–	Violence/homicide	–	Violence/homicide	1 (0.24)	Influenza and pneumonia	37 (1.3)
7	Violence/homicide	–	Other protein-energy malnutrition	3 (17.6)	Bronchitis, emphysema, and asthma	–	Road traffic injuries	3 (0.73)	Violence/homicide	10 (0.3)
8			Tuberculosis	–	Diabetes mellitus	1 (2.1)	Liver diseases	–	Ischemic heart disease	20 (0.7)
9			Stomach cancer	–	Stomach cancer	–	Stomach cancer	–	Cirrhosis and other chronic liver diseases	8 (0.29)
10			Measles	–	Tuberculosis	–	Malnutrition	5 (1.22)	Immuno-preventable diseases	22 (0.8)
11			Violence/homicide	–	Hypertension	–	Other heart diseases (heart failure and arrest)	1 (0.24)	Heart failure, complications and ill-defined diseases	5 (0.18)
12					Nephritis, nephrotic syndrome, and nephrosis	–	Certain conditions originating in the prenatal and perinatal period	10 (2.4)	Urinary system diseases	10 (0.3)
13					Cirrhosis and other chronic liver diseases	–	Urinary system diseases	1 (0.24)	Stomach cancer	4 (0.14)
14					Malnutrition	6 (12.8)			Chronic diseases of the lower respiratory tract	15 (0.5)
15									Certain conditions originating in the prenatal period	24 (0.8)
16									COVID-19	194 (7.0)
n (%)		3 (42.85)		4 (23.53)		12 (25.53)		44 (10.73)		535 (19.50)

### 3.4 International collaboration and distribution by geographic area

**Figure 8:**
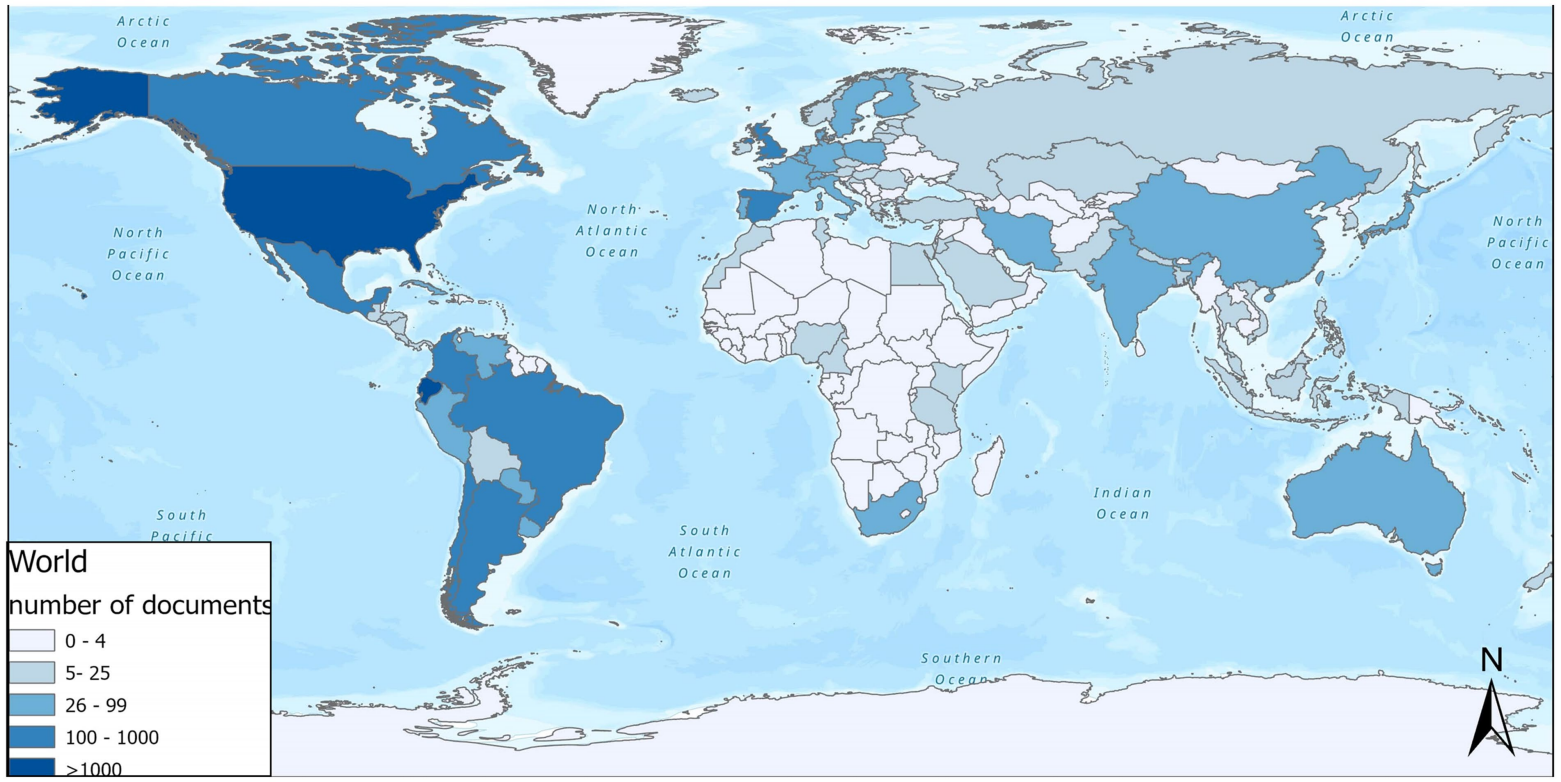
International collaboration according to number of biomedical publications.

**Figure 9:**
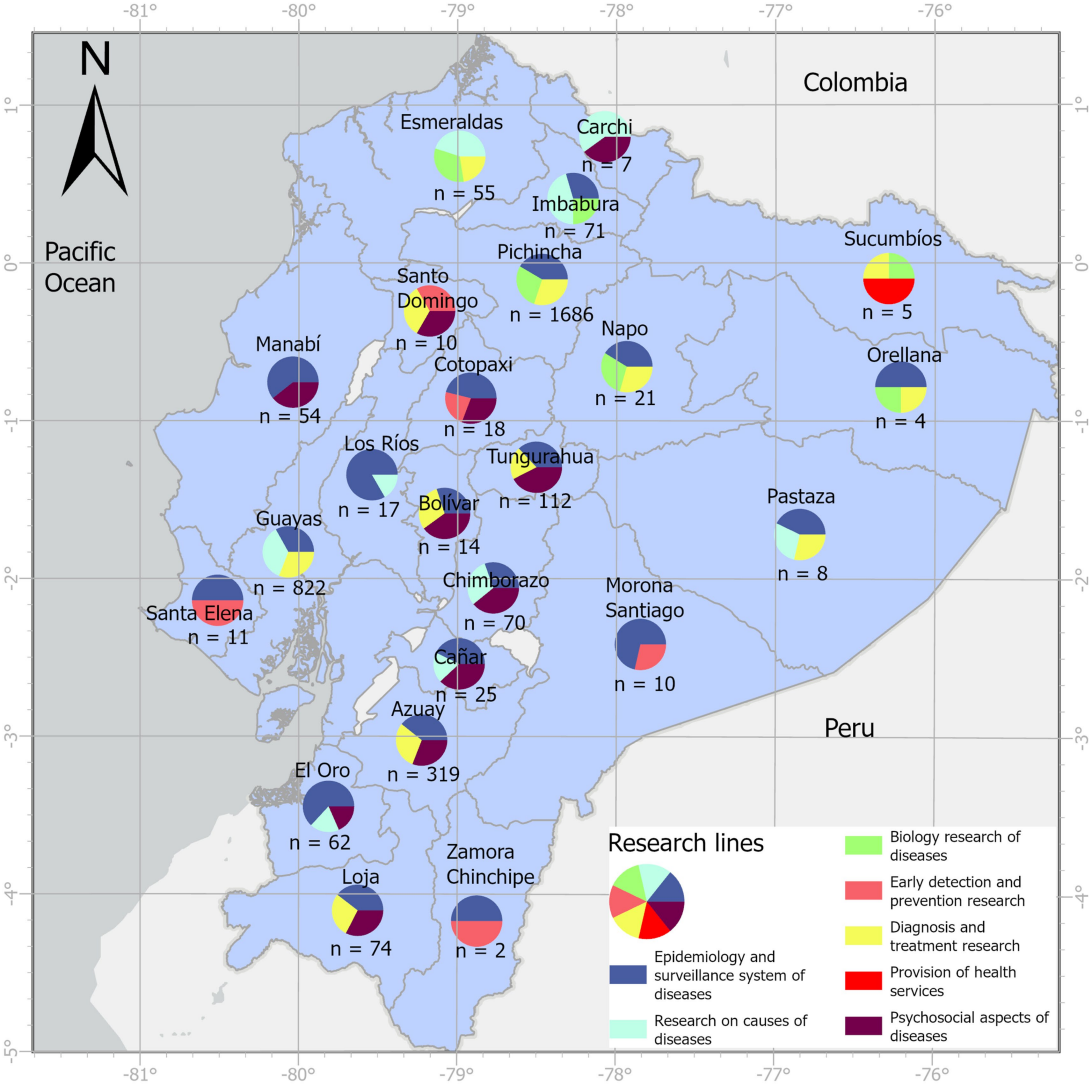
Distribution of Ecuadorian biomedical production by research lines and per province (*n* = 3,225).

Overall, foreign universities/institutions contributed to 70.97% (2,289/3,225) of the scientific output. Ecuadorian researchers collaborated mostly with the following countries: (i) United States (32.77%, *n* = 1,057), (ii) Spain (9.52%, *n* = 307), Brazil (7.63%, *n* = 246), United Kingdom (6.48%, *n* = 209), and Colombia (4.28%, *n* = 138) (Figure 8). The Ecuadorian institutions with the highest number of jointly publications with the United States in descending order are: Universidad San Francisco de Quito USFQ (*n* = 166), MSP (*n* = 160), Universidad Espíritu Santo (*n* = 129), Pontificia Universidad Católica del Ecuador (*n* = 116). This international collaboration has catalyzed research projects in several areas including environmental health due to exposure to pesticides, vector-borne zoonotic diseases, and gastrointestinal pathologies (Supplementary Table S10). Regarding the local distribution of scientific production in biomedicine, we found that the provinces of Pichincha (*n* = 1,686), Guayas (*n* = 822), and Azuay (*n* = 319) are the largest producers of scientific articles, together contributing 87.66% (*n* = 2,827) of the total produced during the period of analysis (Supplementary Figure S5). The focus of research in clinical areas is predominant in the great majority of the 24 provinces of the country, with the exception of Napo province, which registers more research in the area of basic sciences (Supplementary Figure S6). Similarly, the lines of research related to epidemiology and surveillance system of diseases, diagnosis and treatment research, and research on causes of diseases are the most studied at the provincial level (Figure 9).

## 4 Discussion

This research provides an overview of biomedical research in the last century in Ecuador, highlighting the following findings: (i) Exponential increase in scientific production during the last decade, (ii) Predominance of cross-sectional observational studies and studies related to basic biomedical sciences, (iii) Private universities as the largest producers of biomedical research in the country (40.12%), and (iv) Mismatch between the main causes of mortality and local scientific production.

### 4.1 Comparison with other studies

Biomedical research in Ecuador has traditionally been scarce and mostly of a descriptive observational type, until the last decade where an exponential increase in scientific production is evident (16, 22, 23). This increase, we believe, is mainly due to the effect that the LOES has had on the country’s higher education system through the creation of The Council for the Evaluation, Accreditation, and Quality Assurance of Higher Education (CEAACES) in 2011 (16, 22). The CEAACES designed an evaluation model for both public and private universities, one of the parameters being “research” with a weight of 21% of the overall evaluation. Thus, HEIs that had publications indexed in SCImago, Scopus, or WoS received a better evaluation and ranking within the Ecuadorian higher education system (22). This generated a pressure effect on Ecuadorian HEIs and their researchers to increase publication of scientific papers. Among the positive effects of this pressure, local HEIs incorporated mechanisms to stimulate and strengthen research such as hiring research professors with terminal academic degrees (e.g., PhD), reducing teaching hours to dedicate them to research activities, investing in infrastructure such as laboratories, creating internal seed funds, internationalizing policies to attract foreign funds and expertise, and establishing a research culture (23, 24). Additionally, at the government level, funding for research production initially came through the implementation of two programs. The first consisted of a study-abroad grant program for students and faculty members to obtain bachelor and terminal (MS/PhD) degrees in foreign universities. Until 2016 this program benefited about 5,715 Ecuadorian citizens, 21.9% of whom received scholarships in the area of health and wellbeing (25). The second program, called “Prometeo-Viejos Sabios,” was designed to bring foreign researcher expertise to Ecuador to strengthen local research capacity (12). “Prometeo-Viejos Sabios” ended in 2017, but during its execution, it was able to bring 1,046 foreign researchers to Ecuador (8% belonging to health areas) and produced more than 400 documents indexed in Scopus (15). Both programs were available for public and private HEIs. In 2018 the Secretary of Higher Education, Science, Technology, and Innovation (SENESCYT) of Ecuador created a national program for funding research initiatives called “INÉDITA.” This program grants $50,000 USD for individual projects and up to $200,000 USD for collaborative research projects (26). However, despite the positive effects of the LOES/CEAACES to catalyze biomedical research in Ecuador, we also identify negative effects such as a decrease in the performance of studies with higher level of evidence, such as prospective cohorts or randomized clinical trials to inform health policy-making. Several factors could explain this observation including that these types of studies demand greater financial, logistical, and time resources (16). Other contributing factors include inadequate financial investment by the local governments, unstable political environments, ethical and regulatory system obstacles, and the lack of highly/appropriately qualified researchers (4, 16). This decrease in the generation of studies with higher level of evidence has also been documented elsewhere (16, 27). Also, what is surprising is that the same pressure effect exercised by LOES/CEAACES upon local HEIs for publishing has not contributed to addressing the main causes of mortality and morbidity in Ecuador. One example of this finding is that chronic malnutrition in children under 5 years of age continues to be a major public health concern with catastrophic and permanent effects on Ecuadorian children (9). On the other hand, it is important to highlight the high production (17.12%) of local research not related to classic epidemiological designs, which mainly reflects the publication of research at the level of basic sciences in biomedicine. However, compared with neighboring countries such as Colombia, which reported 47.9% of research in the basic sciences during the period 1993–2003, the Ecuadorian production is lower (28). In Ecuador, private universities produce more biomedical research (40.12%) than public universities (19.59%). Also, Ecuadorian private universities produce more research aligned to resolving main causes of local mortality compared to public universities, 38.61% vs. 15.59%, respectively. This lower biomedical production by public universities could be explained by several factors, including: (i) Politicization of public universities. Currently, university authorities (e.g., Chancellors) are appointed through internal elections where even high school graduates are required to vote. This has undermined and interfered with the autonomy, independence, and governance of local public universities. Ultimately, this politicization of the Ecuadorian public higher education system weakens its research endeavors and contributions to national human capital formation and societal development as described in other LMICs (29). (ii) Incapacity to use research funding due to an inefficient, rigid, and highly bureaucratic management system that causes long wait times to acquire basic laboratory supplies or hire well-qualified human resources. For example, Jan Feyen in his publication entitled “WAKE-UP CALL for Ecuador’s universities” describes an administrative hierarchy within public universities through which all activities must pass, resulting in a huge loss of time. He also observes that contracted staff are required to complete activity reports, which are seldom if ever read in the first place (30). (iii) Strong preference for immediate outcomes, which has precluded the realization of long-term and impactful research projects/interventions that assure sustainable funding beyond specific project life cycle. In addition to all this, there are other factors such as the lack of a research culture, overregulation by the state, and lack of funding (31). This has resulted in the fact that, in most cases, private universities in Ecuador are better positioned to conduct research and ranked locally and internationally, according to international rankings such as QS World University Ranking and SCImago Institution Rankings (32, 33). It is important to mention that there are public universities that, despite not having health majors, contribute to local health research, as is the case of ESPOL and YACHAY Tech. This could be explained because ESPOL has undergraduate and graduate programs for the study of biology, nutrition, and biotechnology, and YACHAY Tech has undergraduate programs in biology and biotechnology (34, 35). In general, 18.54% (*n* = 598) of the total produced during the last 100 years in Ecuador is aimed at providing solutions to the main causes of mortality, despite the fact that the MAIS-FCI model, the National Health Research Policy, and the LOES establish that all health research efforts in the country should be aimed at providing solutions to the main causes of mortality (11–13). This percentage decreases to 12.53% when removing publications related to COVID-19, and the mismatch between disease burden and scientific production is more pronounced from the 2000s onwards. This discordance coincides with a local study published in 2021 revealing that only 9% of the scientific production in health sciences was aligned with the main causes of mortality (16). Possible causes for this include a weak national policy and unclear agenda on the country’s health research priorities, weak role of the Ministry of Public Health of Ecuador in monitoring and supporting the articulation of a national health research system, lack of specific competitive funds for these health priorities, lack of appropriately trained researchers, and the absence of cooperation and communication between researchers and key decision makers. In practice, Ecuadorian researchers prioritize other areas and topics that are not aligned with the main health needs of the population, generating a waste of research efforts carried out locally (2, 36).

### 4.2 Strengths and weaknesses

Among the strengths of this research are: (i) Use of well-known and extensive databases such as Scopus and WoS, widely used in bibliometric studies for their quality (17). (ii) The analysis time of 100 years. Previous bibliometric studies conducted in the country have only analyzed up to a maximum of 18 years (16). (iii) Provision of disaggregated information by universities and regions of the country, and international collaboration that was not available in previous studies (16, 27). Limitations of the current work must also be considered. First, we did not consider the regional database Latindex or Redalyc, so publications written in Spanish could have been omitted, which may explain the lack of scientific publications by some universities with health sciences majors. However, due to the broad period analyzed and the use of two comprehensive databases (Scopus and WoS) (17, 18), we anticipate a minimal effect on the final findings. Second, we did not use other metrics or indices besides mortality to assess the impact of local scientific output besides mortality. In future investigations, it might be possible to use other indices such as disability-adjusted life years (36). Third, attributable mortality especially for neglected and/or tropical diseases might be underreported due to errors in national cause-of-death data sets (e.g., INEC), as described elsewhere (37).

### 4.3 Implications of the results

This study provides valuable information for institutions and local decision-makers in charge of formulating and monitoring research and development, higher education, and health policy in Ecuador. One of the findings that was not evident until this study and that calls attention is the low contribution of public universities to research aimed at finding solutions to the main causes of mortality in the country, compared to private universities, 15.59% vs. 38.61%, respectively. Traditionally, public higher education has been considered a central societal resource and public good. In most countries, state-sponsored universities occupy the higher ranks in terms of quality, scientific production, and innovation (38). Public universities are called to use tax money in an efficient, responsible, and ethical manner, and therefore, scientific research produced by public universities should be impactful and of high quality in order to improve population health and health equity (1). However, this is not the case in Ecuador, where, between 1920 and 2021, only 19.59% (632/3,225) of the overall biomedical production was led by public universities, with only 16.72% (100/598) of production aligned to resolving main causes of mortality, compared to 39.97% (239/598) of production by private universities. The Ecuadorian public university should learn from the good practices of its private counterparts in terms of administrative management and research policies (30). On the other hand, the slowdown in the country’s production of intervention studies like clinical trials in the country should call us to reflect and identify its potential causes in addition to those previously mentioned. In Ecuador, there is capacity for high-quality and impactful research; an example of this is the country’s participation in a clinical trial of COVID-19 vaccine published in The New England Journal of Medicine (39). A positive finding that should be supported at all levels is the generation of basic science research in the country; this type of research is essential because the information it provides allows its application in new ways of diagnosing, treating, and preventing diseases (40). In 2010, there was evidence of a consultative effort to define health research priorities in Ecuador; however, there are no evaluation and impact reports available for this activity (41). Future efforts to prioritize what to research in health at the local level should not only focus on the technical and procedural aspects, but also on ethical values such as transparency, inclusion, and accountability (42).

## 5 Conclusion

Biomedical research in Ecuador was first recorded in 1920. Since then, it has been scarce and mainly focused on parasitic infectious diseases and malnutrition. Since 2010 there has been an exponential growth in biomedical publications, which could be due to the effect that the LOES has had on the Ecuadorian higher education system; however, further analysis is needed to evaluate this trend. During the period analyzed (1920–2021), biomedical production was distributed with 52.43% in clinical research, 37.79% in public health, and 9.77% in basic sciences. Private universities are the main generators of biomedical research in the country, and their research efforts are aimed at solving the main causes of local mortality in a higher percentage than their public counterparts. Despite this, there is still a significant mismatch between the burden of disease and local scientific production. Thus, in Ecuador, 18.54% of research produced in a century is destined to solve the main causes of mortality in the country.

## Data availability statement

The raw data supporting the conclusions of this article will be made available by the authors, without undue reservation.

## Author contributions

IS: Writing – review & editing, Writing – original draft, Supervision, Project administration, Methodology, Investigation, Formal analysis, Conceptualization. JC-P: Writing – review & editing, Visualization, Software, Investigation, Formal analysis, Data curation. MCord: Writing – review & editing, Methodology, Investigation, Data curation. CV: Writing – review & editing, Methodology, Investigation, Data curation. MB: Writing – review & editing, Methodology, Investigation, Data curation. MCora: Writing – review & editing, Methodology, Investigation, Data curation. GH-F: Writing – original draft, Formal analysis, Writing – review & editing, Supervision, Methodology, Investigation, Conceptualization.

## Footnotes

^1^ https://www.siies.gob.ec/gra_estadistico_4.html
