# Supplementary material for: One hundred years of Ecuadorian biomedical scientific output and its association with the main causes of mortality: a bibliometric study
Source: Front Med (Lausanne). 2024 Jul 3;11:1395433. doi: 10.3389/fmed.2024.1395433 (PMC11251902; doi:10.3389/fmed.2024.1395433)
Supplement: Supplementary file 1 [file Data_Sheet_1.PDF]

SUPPLEMENTARY MATERIAL

Table of Contents

TABLES……………………………………………………………………………………3

Table S1. Search strategy in Web of Science (WoS) and Scopus…….……………………….3

Table S2. Ecuadorian biomedical sciences-related publications during period I (1920-1944, n=3)..……………………………………………………………………………...7

Table S3. Ecuadorian biomedical sciences-related publications during period II (1945-1969, n=0)……………………………………………………………………………...8

Table S4. Ecuadorian biomedical sciences-related publications during period III (1970-1994, n=35)..……………………………………………..9

Table S5. Ecuadorian biomedical sciences-related publications during period IV (1995-2021, n=3,187)….…………………..…………………………………………………...10

Table S6. Frequency of journals discontinued in Scopus…………………………………11

Table S7. Scientific journals with the highest number of Ecuadorian publications in biomedicine…...14

Table S8. Number of biomedical publications by university and year founded.…...……...15

Table S9. Frequency of biomedical sciences-related publications per institution producer and decade………………………………………………………………………………17

Table S10. Frequency of Ecuadorian institutions with jointly publications with the United States……………………………………………………………………………………17

FIGURES…..……………………………………………………………………………18

Figure S1. Categorization of publications related to the main causes of mortality according to research focus (n=598)………………………..………………………………………18

Figure S2. Distribution of biomedical production related to the main causes of mortality by higher education institutions after removing COVID-19 related publications…………….19

Figure S3. Distribution of INEC studies without COVID-19-related articles by producing institution (n=404))………………………..……………………………………………20

Figure S4. Time trend of Ecuadorian biomedical sciences-related publications aligned with main causes of mortality according to the INEC by institution producer (n=598)………..21

Figure S5. Number of articles per province (N = 3,225)..…………………..…………….22

Figure S6. Research focus per province (N = 3,225)……………………………………...23

Table S1. Search strategy in Web of Science (WoS) and Scopus

Database	Equation
**WoS**	https://www.webofscience.com/wos/woscc/summary/9e7fa568-e2c9-4bbd-9520-b1852537925e-9a6abfff/relevance/1
**Scopus**	AFFILCOUNTRY(Ecuador) AND (EXCLUDE (PUBYEAR,2023) OR EXCLUDE (PUBYEAR,2022)) AND (LIMIT-TO (SUBJAREA,"MEDI") OR LIMIT-TO (SUBJAREA,"AGRI") OR LIMIT-TO (SUBJAREA,"ENVI") OR LIMIT-TO (SUBJAREA,"BIOC") OR LIMIT-TO (SUBJAREA,"IMMU") OR LIMIT-TO (SUBJAREA,"ECON") OR LIMIT-TO (SUBJAREA,"PHAR") OR LIMIT-TO (SUBJAREA,"NEUR") OR LIMIT-TO (SUBJAREA,"MULT") OR LIMIT-TO (SUBJAREA,"VETE") OR LIMIT-TO (SUBJAREA,"PSYC") OR LIMIT-TO (SUBJAREA,"NURS") OR LIMIT-TO (SUBJAREA,"HEAL") OR LIMIT-TO (SUBJAREA,"DENT")) AND (LIMIT-TO (EXACTKEYWORD,"Human") OR LIMIT-TO (EXACTKEYWORD,"Article") OR LIMIT-TO (EXACTKEYWORD,"Ecuador") OR LIMIT-TO (EXACTKEYWORD,"Humans") OR LIMIT-TO (EXACTKEYWORD,"Female") OR LIMIT-TO (EXACTKEYWORD,"Male") OR LIMIT-TO (EXACTKEYWORD,"Adult") OR LIMIT-TO (EXACTKEYWORD,"Controlled Study") OR LIMIT-TO (EXACTKEYWORD,"Priority Journal") OR LIMIT-TO (EXACTKEYWORD,"Nonhuman") OR LIMIT-TO (EXACTKEYWORD,"Major Clinical Study") OR LIMIT-TO (EXACTKEYWORD,"Animal") OR LIMIT-TO (EXACTKEYWORD,"Middle Aged") OR LIMIT-TO (EXACTKEYWORD,"Aged") OR LIMIT-TO (EXACTKEYWORD,"Child") OR LIMIT-TO (EXACTKEYWORD,"Adolescent") OR LIMIT-TO (EXACTKEYWORD,"Genetics") OR LIMIT-TO (EXACTKEYWORD,"Prevalence") OR LIMIT-TO (EXACTKEYWORD,"Risk Factor") OR LIMIT-TO (EXACTKEYWORD,"Procedures") OR LIMIT-TO (EXACTKEYWORD,"Review") OR LIMIT-TO (EXACTKEYWORD,"Cross-sectional Study") OR LIMIT-TO (EXACTKEYWORD,"Physiology") OR LIMIT-TO (EXACTKEYWORD,"Clinical Article") OR LIMIT-TO (EXACTKEYWORD,"Metabolism") OR LIMIT-TO (EXACTKEYWORD,"Chemistry") OR LIMIT-TO (EXACTKEYWORD,"Case Report") OR LIMIT-TO (EXACTKEYWORD,"Unclassified Drug") OR LIMIT-TO (EXACTKEYWORD,"Young Adult") OR LIMIT-TO (

	EXACTKEYWORD,"Andes") OR LIMIT-TO (EXACTKEYWORD,"Phylogeny") OR LIMIT-TO (EXACTKEYWORD,"Questionnaire") OR LIMIT-TO (EXACTKEYWORD,"Classification") OR LIMIT-TO (EXACTKEYWORD,"Risk Factors") OR LIMIT-TO (EXACTKEYWORD,"Cross-Sectional Studies") OR LIMIT-TO (EXACTKEYWORD,"Mortality") OR LIMIT-TO (EXACTKEYWORD,"Epidemiology") OR LIMIT-TO (EXACTKEYWORD,"Isolation And Purification") OR LIMIT-TO (EXACTKEYWORD,"Child, Preschool") OR LIMIT-TO (EXACTKEYWORD,"Comparative Study") OR LIMIT-TO (EXACTKEYWORD,"Infant") OR LIMIT-TO (EXACTKEYWORD,"Preschool Child") OR LIMIT-TO (EXACTKEYWORD,"Taxonomy") OR LIMIT-TO (EXACTKEYWORD,"Risk Assessment") OR LIMIT-TO (EXACTKEYWORD,"Microbiology") OR LIMIT-TO (EXACTKEYWORD,"Animal Experiment") OR LIMIT-TO (EXACTKEYWORD,"Clinical Trial") OR LIMIT-TO (EXACTKEYWORD,"Pregnancy") OR LIMIT-TO (EXACTKEYWORD,"COVID-19") OR LIMIT-TO (EXACTKEYWORD,"Galapagos Islands") OR LIMIT-TO (EXACTKEYWORD,"Polymerase Chain Reaction") OR LIMIT-TO (EXACTKEYWORD,"Treatment Outcome") OR LIMIT-TO (EXACTKEYWORD,"Drug Effect") OR LIMIT-TO (EXACTKEYWORD,"Age") OR LIMIT-TO (EXACTKEYWORD,"Disease Severity") OR LIMIT-TO (EXACTKEYWORD,"Cohort Analysis") OR LIMIT-TO (EXACTKEYWORD,"Disease Association") OR LIMIT-TO (EXACTKEYWORD,"Ecosystem") OR LIMIT-TO (EXACTKEYWORD,"Genotype") OR LIMIT-TO (EXACTKEYWORD,"Letter") OR LIMIT-TO (EXACTKEYWORD,"Parasitology") OR LIMIT-TO (EXACTKEYWORD,"Clinical Feature") OR LIMIT-TO (EXACTKEYWORD,"Public Health") OR LIMIT-TO (EXACTKEYWORD,"Retrospective Study") OR LIMIT-TO (EXACTKEYWORD,"Hypertension") OR LIMIT-TO (EXACTKEYWORD,"Blood") OR LIMIT-TO (EXACTKEYWORD,"Human Experiment") OR LIMIT-TO (EXACTKEYWORD,"Aged, 80 And Over") OR LIMIT-TO (EXACTKEYWORD,"Pathology") OR LIMIT-TO (EXACTKEYWORD,"Obesity") OR LIMIT-TO (EXACTKEYWORD,"Prospective Study") OR LIMIT-TO (EXACTKEYWORD,"Incidence") OR LIMIT-TO (EXACTKEYWORD,"Nuclear Magnetic Resonance Imaging") OR LIMIT-TO (EXACTKEYWORD,"Demography") OR LIMIT-TO (

	EXACTKEYWORD,"Coronavirus Disease 2019") OR LIMIT-TO (EXACTKEYWORD,"Human Tissue") OR LIMIT-TO (EXACTKEYWORD,"Computer Assisted Tomography") OR LIMIT-TO (EXACTKEYWORD,"Animalia") OR LIMIT-TO (EXACTKEYWORD,"Body Mass") OR LIMIT-TO (EXACTKEYWORD,"Genetic Variability") OR LIMIT-TO (EXACTKEYWORD,"Rural Population") OR LIMIT-TO (EXACTKEYWORD,"Complication") OR LIMIT-TO (EXACTKEYWORD,"Psychology") OR LIMIT-TO (EXACTKEYWORD,"Pathophysiology") OR LIMIT-TO (EXACTKEYWORD,"Very Elderly") OR LIMIT-TO (EXACTKEYWORD,"Growth, Development And Aging") OR LIMIT-TO (EXACTKEYWORD,"Quality Of Life") OR LIMIT-TO (EXACTKEYWORD,"Morphology") OR LIMIT-TO (EXACTKEYWORD,"Multicenter Study") OR LIMIT-TO (EXACTKEYWORD,"Diet") OR LIMIT-TO (EXACTKEYWORD,"Newborn") OR LIMIT-TO (EXACTKEYWORD,"Geographic Distribution") OR LIMIT-TO (EXACTKEYWORD,"Phenotype") OR LIMIT-TO (EXACTKEYWORD,"School Child") OR LIMIT-TO (EXACTKEYWORD,"Drug Efficacy") OR LIMIT-TO (EXACTKEYWORD,"Observational Study") OR LIMIT-TO (EXACTKEYWORD,"Health Survey") OR LIMIT-TO (EXACTKEYWORD,"Anura") OR LIMIT-TO (EXACTKEYWORD,"Rural Area") OR LIMIT-TO (EXACTKEYWORD,"Amazonia") OR LIMIT-TO (EXACTKEYWORD,"Aves") OR LIMIT-TO (EXACTKEYWORD,"Species Diversity") OR LIMIT-TO (EXACTKEYWORD,"Human Cell") OR LIMIT-TO (EXACTKEYWORD,"Sex Difference") OR LIMIT-TO (EXACTKEYWORD,"Genetic Variation") OR LIMIT-TO (EXACTKEYWORD,"Species Richness") OR LIMIT-TO (EXACTKEYWORD,"Nucleotide Sequence") OR LIMIT-TO (EXACTKEYWORD,"Pandemic") OR LIMIT-TO (EXACTKEYWORD,"Education") OR LIMIT-TO (EXACTKEYWORD,"Diabetes Mellitus") OR LIMIT-TO (EXACTKEYWORD,"Immunology") OR LIMIT-TO (EXACTKEYWORD,"Randomized Controlled Trial") OR LIMIT-TO (EXACTKEYWORD,"Prospective Studies") OR LIMIT-TO (EXACTKEYWORD,"Plant Extract") OR LIMIT-TO (EXACTKEYWORD,"Virology") OR LIMIT-TO (EXACTKEYWORD,"Decision Making") OR LIMIT-TO (EXACTKEYWORD,"Depression") OR LIMIT-TO (EXACTKEYWORD,"Methodology") OR LIMIT-TO (EXACTKEYWORD,"Sensitivity And Specificity") OR LIMIT-TO (

	EXACTKEYWORD,"New Species") OR LIMIT-TO (EXACTKEYWORD,"Practice Guideline") OR LIMIT-TO (EXACTKEYWORD,"Surveys And Questionnaires") OR LIMIT-TO (EXACTKEYWORD,"DNA Sequence") OR LIMIT-TO (EXACTKEYWORD,"Enzyme Linked Immunosorbent Assay") OR LIMIT-TO (EXACTKEYWORD,"Physical Activity") OR LIMIT-TO (EXACTKEYWORD,"Animal Tissue") OR LIMIT-TO (EXACTKEYWORD,"Biomass") OR LIMIT-TO (EXACTKEYWORD,"Infant, Newborn") OR LIMIT-TO (EXACTKEYWORD,"Diagnostic Imaging") OR LIMIT-TO (EXACTKEYWORD,"In Vitro Study") OR LIMIT-TO (EXACTKEYWORD,"SARS-CoV-2") OR LIMIT-TO (EXACTKEYWORD,"Antiinfective Agent") OR LIMIT-TO (EXACTKEYWORD,"Escherichia Coli") OR LIMIT-TO (EXACTKEYWORD,"Statistics And Numerical Data") OR LIMIT-TO (EXACTKEYWORD,"Socioeconomics") OR LIMIT-TO (EXACTKEYWORD,"Neurocysticercosis") OR LIMIT-TO (EXACTKEYWORD,"Environmental Monitoring") OR LIMIT-TO (EXACTKEYWORD,"Prediction") OR LIMIT-TO (EXACTKEYWORD,"Retrospective Studies") OR LIMIT-TO (EXACTKEYWORD,"Statistical Model") OR LIMIT-TO (EXACTKEYWORD,"Altitude") OR LIMIT-TO (EXACTKEYWORD,"Environmental Protection")) AND (LIMIT-TO (DOCTYPE,"ar"))

Table S2. Ecuadorian biomedical sciences-related publications during period I (1920-1944, n=3)

Characteristic	Public University n= 1	Private University n= 0	Mixed University n= 0	Non-academic Institution n= 2
**SCImago Journal Rank, n(%)**				
**Q1**	0	0	0	2 (100)
**Q2**	0	0	0	0
**Q3**	0	0	0	0
**Q4**	0	0	0	0
**None**	1 (100)	0	0	0
**Study design, n(%)**				
**Case/series report**	1 (100)	0	0	0
**Ecological**	0	0	0	0
**Cross-sectional/survey**	0	0	0	0
**Case-control**	0	0	0	0
**Cohort**	0	0	0	1 (50)
**Randomized controlled trial**	0	0	0	0
**Systematic review**	0	0	0	0
**Meta-analysis**	0	0	0	0
**Other**	0	0	0	1 (50)
**Research focus, n(%)**				
**Basic**	0	0	0	0
**Clinical**	1 (100)	0	0	1 (50)
**Public Health**	0	0	0	1 (50)

Table S3. Ecuadorian biomedical sciences-related publications during period II (1945-1969, n=0)

Characteristic	Public University n= 0	Private University n= 0	Mixed University n= 0	Non-academic Institution n= 0
**SCImago Journal Rank, n(%)**				
**Q1**	0	0	0	0
**Q2**	0	0	0	0
**Q3**	0	0	0	0
**Q4**	0	0	0	0
**None**	0	0	0	0
**Study design, n(%)**				
**Case/series report**	0	0	0	0
**Ecological**	0	0	0	0
**Cross-sectional/survey**	0	0	0	0
**Case-control**	0	0	0	0
**Cohort**	0	0	0	0
**Randomized controlled trial**	0	0	0	0
**Systematic review**	0	0	0	0
**Meta-analysis**	0	0	0	0
**Other**	0	0	0	0
**Research focus, n(%)**				
**Basic**	0	0	0	0
**Clinical**	0	0	0	0
**Public Health**	0	0	0	0

Table S4. Ecuadorian biomedical sciences-related publications during period III (1970-1994, n=35)

Characteristic	Public University n= 18	Private University n= 2	Mixed University n= 0	Non-academic Institution n= 15
**SCImago journal rank, n(%)**				
**Q1**	13 (72.22)	1 (50)	0	9 (60)
**Q2**	4 (22.22)	0	0	2 (13.33)
**Q3**	0	1 (50)	0	2 (13.33)
**Q4**	0	0	0	1 (6.67)
**None**	1 (5.56)	0	0	1 (6.67)
**Study design, n(%)**				
**Case/series report**	0	0	0	0
**Ecological**	0	0	0	0
**Cross-sectional/survey**	5 (27.78)	2 (100)	0	10 (66.67)
**Case-control**	3 (16.67)	0	0	0
**Cohort**	1 (5.56)	0	0	0
**Randomized controlled trial**	2 (11.11)	0	0	0
**Systematic review**	0	0	0	3 (20)
**Meta-analysis**	0	0	0	0
**Other**	7 (38.89)	0	0	2 (13.33)
**Research focus, n(%)**				
**Basic**	7 (38.89)	1 (50)	0	0
**Clinical**	10 (55.56)	0	0	6 (40)
**Public Health**	1 (5.56)	1 (50)	0	9 (60)

Table S5. Ecuadorian biomedical sciences-related publications during period IV (1995-2021, n=3,187)

Characteristic	Public University n= 613	Private University n= 1292	Mixed University n= 331	Non-academic Institution n= 951	p-value*
**SCImago journal rank, n(%)**					<0.001
**Q1**	226 (36.87)	537 (41.56)	119 (35.95)	490 (51.52)	
**Q2**	130 (21.21)	296 (22.91)	79 (23.87)	227 (23.87)	
**Q3**	90 (16.68)	173 (13.39)	37 (11.18)	118 (12.41)	
**Q4**	152 (24.80)	238 (18.42)	87 (26.28)	101 (10.62)	
**None**	15 (2.45)	48 (3.72)	9 (2.72)	15 (1.58)	
**Study design, n(%)**					
**Case/series report**	2 (0.33)	6 (0.46)	3 (0.91)	6 (0.63)	-
**Ecological**	20 (3.26)	23 (1.78)	9 (2.72)	100 (10.52)	
**Cross-sectional/survey**	326 (53.18)	674 (52.17)	187 (56.50)	398 (41.85)	
**Case-control**	20 (3.26)	80 (6.19)	19 (5.74)	13 (1.37)	
**Cohort**	50 (8.16)	253 (19.58)	36 (10.88)	163 (17.14)	
**Randomized controlled trial**	33 (5.38)	27 (2.09)	3 (0.91)	49 (5.15)	
**Systematic review**	24 (3.92)	40 (3.10)	9 (2.72)	61 (6.41)	
**Meta-analysis**	1 (0.16)	2 (0.15)	0	8 (0.84)	
**Other**	137 (22.35)	187 (14.47)	65 (19.64)	153 (16.09)	
**Research focus, n(%)**					<0.001
**Basic**	64 (10.44)	139 (10.76)	38 (11.48)	66 (6.94)	
**Clinical**	286 (46.66)	719 (55.65)	171 (51.66)	497 (52.26)	
**Public Health**	263 (42.90)	434 (33.59)	122 (36.86)	388 (40.80)	

*Categorical data was analyzed using chi-square test.

Table S6. Frequency of journals discontinued in Scopus

Journal name	Year of discontinuation
Archivos Venezolanos de Farmacologia y Terapeutica	2022
Ciencia UNEMI	NA
Acta Bioquimica Clinica Latinoamericana	2017
Revista Publicando	NA
Universitas-Revista De Ciencias Sociales Y Humanas	NA
Cureus	NA
Pharmacologyonline	NA
Revista San Gregorio	NA
American Journal of Physical Anthropology	2021
Australasian Medical Journal	2017
International Journal of Occupational and Environmental Health	2018
Revista Ciencias Pedagogicas e Innovacion	NA
Acta Psiquiatrica y Psicologica de America Latina	1996
Alteridad-Revista de Educacion	NA
Asian Journal of Pharmaceutical and Clinical Research	2018
Asian Journal of Pharmaceutics	2018
Avances En Biomedicina	NA
BMC Dermatology	2020
BMC International Health and Human Rights	2020
Boletin de la Oficina Sanitaria Panamericana	1996
British Journal of Obstetrics and Gynaecology	2001
Circulation	NA
Cirugia y Cirujanos (English Edition)	NA
Duazary	NA

International Congress Series	2007
Omega (United States)	NA
Psicodebate-Psicologia Cultura y Sociedad	NA
Psicologia Conocimiento y Sociedad	NA
Revista Brasileira de Cardiologia Invasiva	2016
Revista Cientifica de la Facultad de Ciencias Veterinarias de la Universidad del Zulia	NA
Revista Iberoamericana de Diagnostico y Evaluacion-E Avaliacao Psicologica	NA
Revista Mexicana de Cardiologia	2018
African Journal of Traditional, Complementary, and Alternative Medicines: AJTCAM	2017
American Trypanosomiasis Chagas Disease: One Hundred Years of Research	NA
Annals of Tropical Medicine and Parasitology	2011
Archives of Ophthalmology	2013
Archivos de Medicina	2015
Archivos de Medicina Veterinaria	2016
Ascaris: The Neglected Parasite	NA
BMC Evolutionary Biology	2020
BMC Medical Genetics	2020
Boletin de Pediatria	NA
Campus Virtuales	2022
Cancer	NA
Clujul Medical	2018
Community Genetics	2008
Cuadernos De Medicina Forense	2018
Drug Information Journal	2013
Endothelium-Journal of Endothelial Cell Research	2008
Entomology and Applied Science Letters	NA
Environmental Challenges in The Pacific Basin	NA
Eureka-Revista Cientifica de Psicologia	NA
Foro Educacional	NA

Gabi Journal-Generics and Biosimilars Initiative Journal	NA
Genome Announcements	2018
Global Journal of Health Science	2016
Ibero-American Bioethics: History and Perspectives	NA
International Encyclopedia of Public Health, Vol 1, A-Chl, 2nd Edition	NA
International Journal of Osteoporosis and Metabolic Disorders	2018
Internet Journal of Pulmonary Medicine	2015
Journal Of Negative Results in Biomedicine	2017
Mutation Research-Environmental Mutagenesis and Related Subjects	1996
Pensando Psicologia	NA
Pragmatic And Observational Research	NA
Psiencia-Revista Latinoamericana de Ciencia Psicologica	NA
Punto Genero	NA
Revista Chilena de Pediatria-Chile	2021
Revista Colombiana de Cancerologia	NA
Revista Colombiana de Ciencias Sociales	NA
Revista Cubana de Reumatologia	NA
Revista de Cirugia	NA
Revista Medica del Instituto Mexicano del Seguro Social	NA
Salud Arte y Cuidado	NA
Smart Technology Applications in Business Environments	NA
Tropical Veterinary Diseases: Control And Prevention in the Context of the New World Order	NA
Who Chronicle	NA

NA= not applicable because these journals are part of the WoS database.

Table S7. Scientific journals with the highest number of Ecuadorian publications in biomedicine

Journal name	Country	n (%)	QW	QS
**Archivos Venezolanos de Farmacología y Terapéutica**	Venezuela	89 (2.76)	-	Q4
**Revista Ecuatoriana de Neurología**	Ecuador	76 (2.36)	-	Q4
**American Journal of Tropical Medicine and Hygiene**	United States	65 (2.01)	Q2	Q2
**PLOS One**	United States	64 (1.98)	Q2	Q1
**International Journal of Environmental Research and Public Health**	Switzerland	63 (1.95)	Q2	Q1
**PLOS Neglected Tropical Diseases**	United States	49 (0.99)	Q1	Q1
**Pan American Journal of Public Health**	United States	32 (1.45)	Q3	Q2
**Revista Latinoamericana de Hipertensión**	Venezuela	38 (1.17)	-	Q3
**Forensic Science International: Genetics Supplement Series**	Ireland	30 (0.93)	-	Q4
**Investigación Clínica**	Venezuela	31 (0.96)	-	Q4

QW, Quartile WoS; QS, Quartile Scopus.

Tabla S8. Number and rate of biomedical publications by university and year founded

University (acronyms)	n	City	Type	Under	D. F.	Spe	MSc	PhD	Articles per 1,000 students
Pontificia Universidad Católica Del Ecuador (PUCE)	357	Quito	Mixed	✓	1994	✓	✓	-	47.35
Universidad San Francisco De Quito (USFQ)	354	Quito	Private	✓	1994	✓	✓	-	295.56
Universidad Central Del Ecuador (UCE)	342	Quito	Public	✓	1835	✓	-	-	41.10
Universidad Espíritu Santo (UEES)	288	Guayaquil	Private	✓	2007	✓	✓	-	239.59
Universidad de las Américas (UDLA)	225	Quito	Private	✓	ND.	✓	✓	-	48.07
Universidad de Cuenca (UC)	215	Cuenca	Public	✓	1968	✓	✓	-	48.71
Universidad Católica De Santiago De Guayaquil (UCSG)	179	Guayaquil	Mixed	✓	1968	✓	✓	-	27.55
Universidad Tecnológica Equinoccial (UTE)	104	Quito	Mixed	✓	1986	✓	✓	-	56.73
Universidad de Guayaquil (UG)	104	Guayaquil	Public	✓	1877	✓	✓	-	13.05
Universidad Católica de Cuenca (UCACUE)	103	Cuenca	Mixed	✓	2003	✓	✓	-	11.21
Escuela Superior Politécnica del Litoral (ESPOL)	76	Guayaquil	Public	-	-	-	-	-	737.86
Universidad Técnica Particular de Loja (UTPL)	66	Loja	Mixed	✓	2000	✓	✓	-	38.26
Universidad de las Fuerzas Armadas (ESPE)	63	Sangolquí	Public	✓	2019	-	✓	-	-
Universidad Regional Autónoma de los Andes (UNIANDES)	56	Ambato	Private	✓	1997	-	✓	-	14.65
Universidad Técnica de Ambato (UTA)	52	Ambato	Public	✓	ND.	-	✓	-	14.55
Universidad Internacional del Ecuador (UIDE)	48	Quito	Private	✓	2002	-	✓	-	65.63
Universidad Técnica de Machala (UTMACH)	42	Machala	Public	✓	1970	✓	-	-	16.61
Escuela Superior Politécnica del Chimborazo (ESPOCH)	40	Riobamba	Public	✓	1972	-	✓	-	11.75
Universidad del Azuay (UAZUAY)	34	Cuenca	Mixed	✓	ND.	-	-	-	45.38
Universidad Politécnica Salesiana (UPS)	33	Cuenca	Mixed	✓	-	-	-	-	-

Universidad de Investigación de Tecnología Experimental Yachay	33	Urcuquí	Public	-	-	-	-	-	-
Escuela Politécnica Nacional (EPN)	31	Quito	Public	-	-	-	-	-	-
Universidad Nacional de Chimborazo (UNACH)	28	Riobamba	Public	✓	ND.	-	✓	-	9.56
Universidad Técnica de Manabí (UTM)	26	Portoviejo	Public	✓	1991	✓	✓	-	5.65
Universidad Técnica del Norte (UTN)	23	Ibarra	Public	✓	1986	-	✓	-	13.60
Universidad Tecnológica Indoamérica (UTI)	20	Ambato	Private	✓	ND.	-	-	-	-
Universidad Laica Eloy Alfaro de Manabí (ULEAM)	17	Manta	Public	✓	1979	✓	-	-	6.06
Universidad Internacional SEK (UISEK)	15	Quito	Private	✓	2007	-	✓	-	-
Universidad Estatal de Milagro (UNEMI)	13	Milagro	Public	✓	ND.	-	-	-	5.26
Universidad Regional Amazónica IKIAM (IKIAM)	12	Tena	Public	-	-	-	-	-	-
Universidad Estatal de Bolívar (UEB)	9	Guanujo	Public	✓	1989	-	-	-	11.04
Universidad Andina Simón Bolívar (UASB)	9	Quito	Public	✓	ND.	-	✓	✓	64.17
Facultad Latinoamericana de Ciencias Sociales (FLACSO)	8	Quito	Private	-	-	-	-	-	-
Universidad Nacional de Loja (UNL)	5	Loja	Public	✓	1969	✓	-	-	2.51
Universidad Estatal Península de Santa Elena (UPSE)	5	La Libertad	Public	✓	ND.	-	-	-	8.42
Universidad Técnica Estatal de Quevedo (UTEQ)	4	Quevedo	Public	✓	ND.	-	-	-	0.04
Universidad Estatal Amazónica (UEA)	4	Puyo	Private	-	-	-	-	-	-
Universidad Técnica de Cotopaxi (UTC)	3	Latacunga	Public	-	-	-	-	-	-
Universidad Nacional de Educación Ecuador (UNAE)	3	Chuquipata	Public	-	-	-	-	-	-
Universidad Metropolitana del Ecuador (UMET)	3	Guayaquil	Private	✓	-	-	✓	-	2.28
Universidad Tecnológica ECOTEC (ECOTEC)	2	Guayaquil	Private	✓	ND.	-	-	-	16.27
Universidad Tecnológica Israel (UISRAEL)	1	Quito	Private	-	ND.	-	-	-	-

✓ (Yes), - (No), ND. (No Data), Under. (Undergraduate), D.F (Date founded), Spe. (Specialization), MSc. (Master's degree), PhD. (Doctor of Philosophy).

Table S9. Frequency of biomedical sciences-related publications per institution producer and decade

Type of institution	<1980 n=7	1980-1989 n=17	1990-1999 n=47	2000-2010 n=410	2011-2021 n=2744
Public university	2	9	22	57	542
Private university	0	1	6	116	1171
Mixed (Public & private university)	0	0	1	20	310
Non-academic	5	7	18	217	721

Table S10. Frequency of Ecuadorian institutions with jointly publications with the United States

Name of the institution	Number of articles
Universidad San Francisco de Quito	166
Ministerio de Salud	160
Universidad Espíritu Santo	129
Pontificia Universidad Católica del Ecuador	116
Universidad Central del Ecuador	57
Universidad de las Américas	47
Instituto Nacional Investigación de Salud Pública	43
Universidad de Cuenca	24
Universidad Católica de Santiago de Guayaquil	21
Universidad Técnica de Machala	12
Universidad Internacional del Ecuador	11

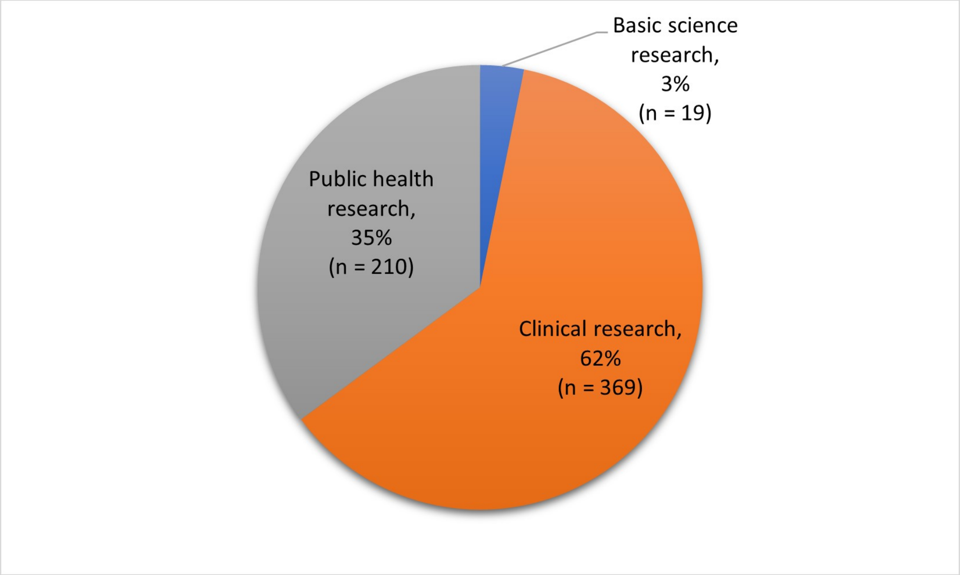

Figure S1. Categorization of publications related to the main causes of mortality according to research focus (n=598)

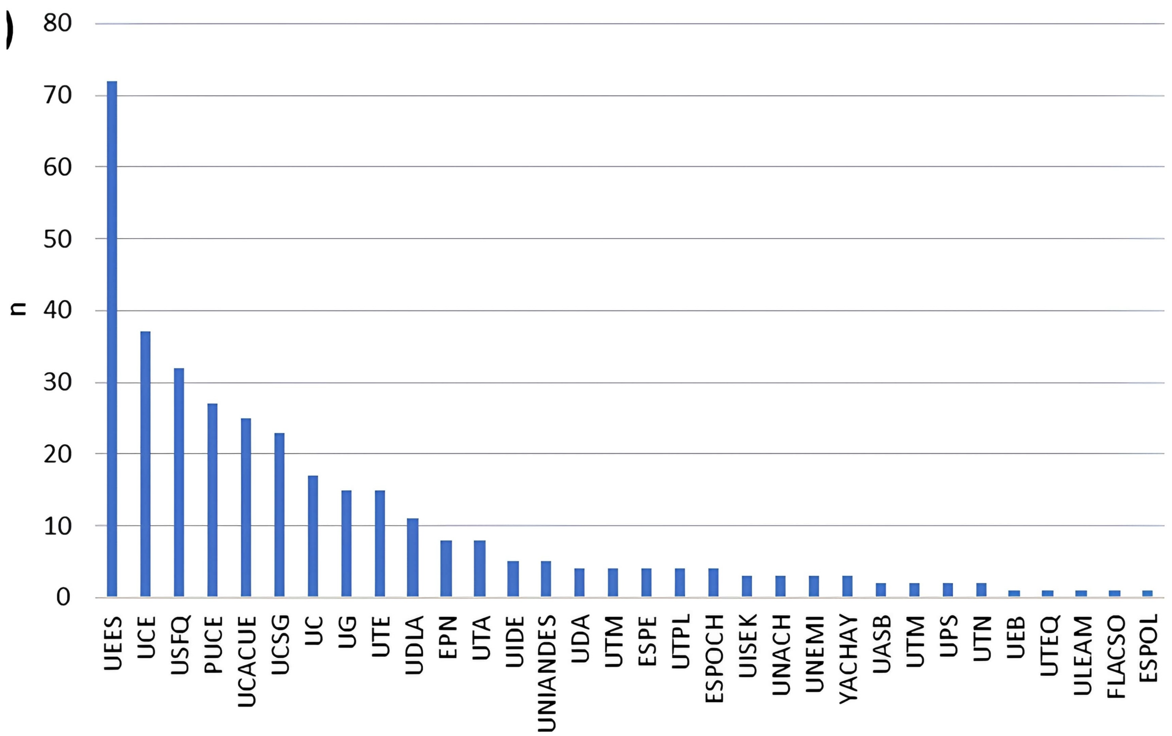

Figure S2. Distribution of biomedical production related to the main causes of mortality by higher education institutions after removing COVID-19 related publications.

Abbreviations: UEES, Universidad Espíritu Santo; UDLA, Universidad de las Américas; UCE, Universidad Central del Ecuador; PUCE, Pontificia Universidad Católica del Ecuador; USFQ, Universidad San Francisco de Quito; UCACUE, Universidad Católica de Cuenca; UCSG, Universidad Católica Santiago de Guayaquil; UC, Universidad de Cuenca; UG, Universidad de Guayaquil; UTE, Universidad Tecnológica Equinoccial; UIDE, Universidad Internacional del Ecuador; UNIANDES, Universidad Regional Autónoma de los Andes; ESPOCH, Escuela Superior Politécnica de Chimborazo; UTA, Universidad Técnica de Ambato; UDA, Universidad del Azuay; UTM, Universidad Técnica de Manabí; ESPE, Universidad de las Fuerzas Armadas; UTPL, Universidad Técnica Particular de Loja; UISEK, Universidad Internacional SEK; UNACH, Universidad Nacional de Chimborazo; UNEMI, Universidad Estatal de Milagro; Yachay, Universidad de Investigación de Tecnología Experimental Yachay; UASB, Universidad Andina Simón Bolívar; UTM, Universidad Técnica de Manabí; UPS, Universidad Politécnica Salesiana; UTN, Universidad Técnica del Norte; UEB, Universidad Estatal de Bolívar; UTEQ, Universidad Técnica de Quevedo; ULEAM, Universidad Laica Eloy Alfaro de Manabí; UTB, Universidad Técnica de Babahoyo; ECOTEC, Universidad Tecnológica ECOTEC; UNAE, Universidad Nacional de Educación; FLACSO, Facultad Latinoamericana de Ciencias Sociales; UTC, Universidad Técnica de Cotopaxi; ESPOL, Escuela Superior Politécnica del Litoral.

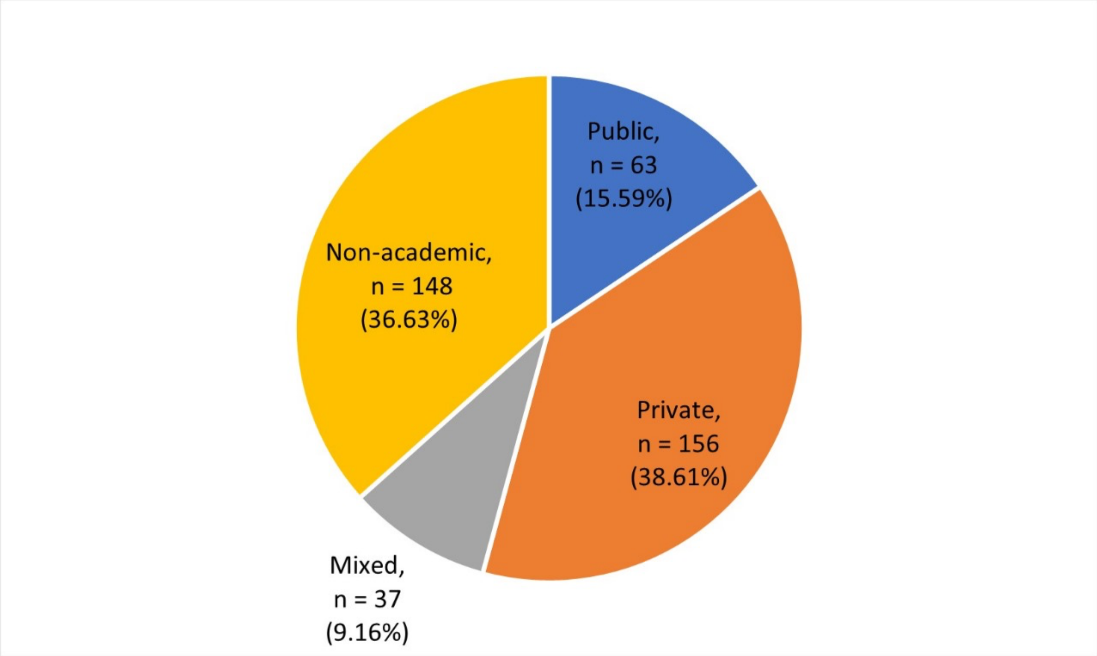

Figure S3. Distribution of INEC studies without COVID-19-related articles by producing institution (n=404)

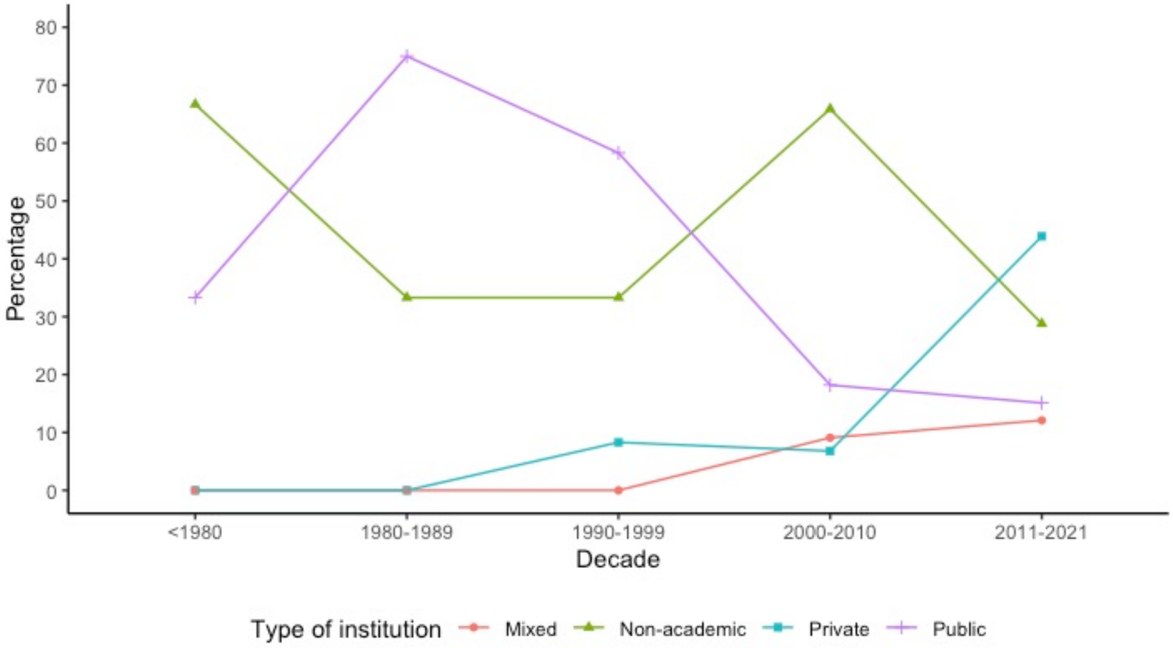

Figure S4. Time trend of Ecuadorian biomedical sciences-related publications aligned with main causes of mortality according to the INEC by institution producer (n=598)

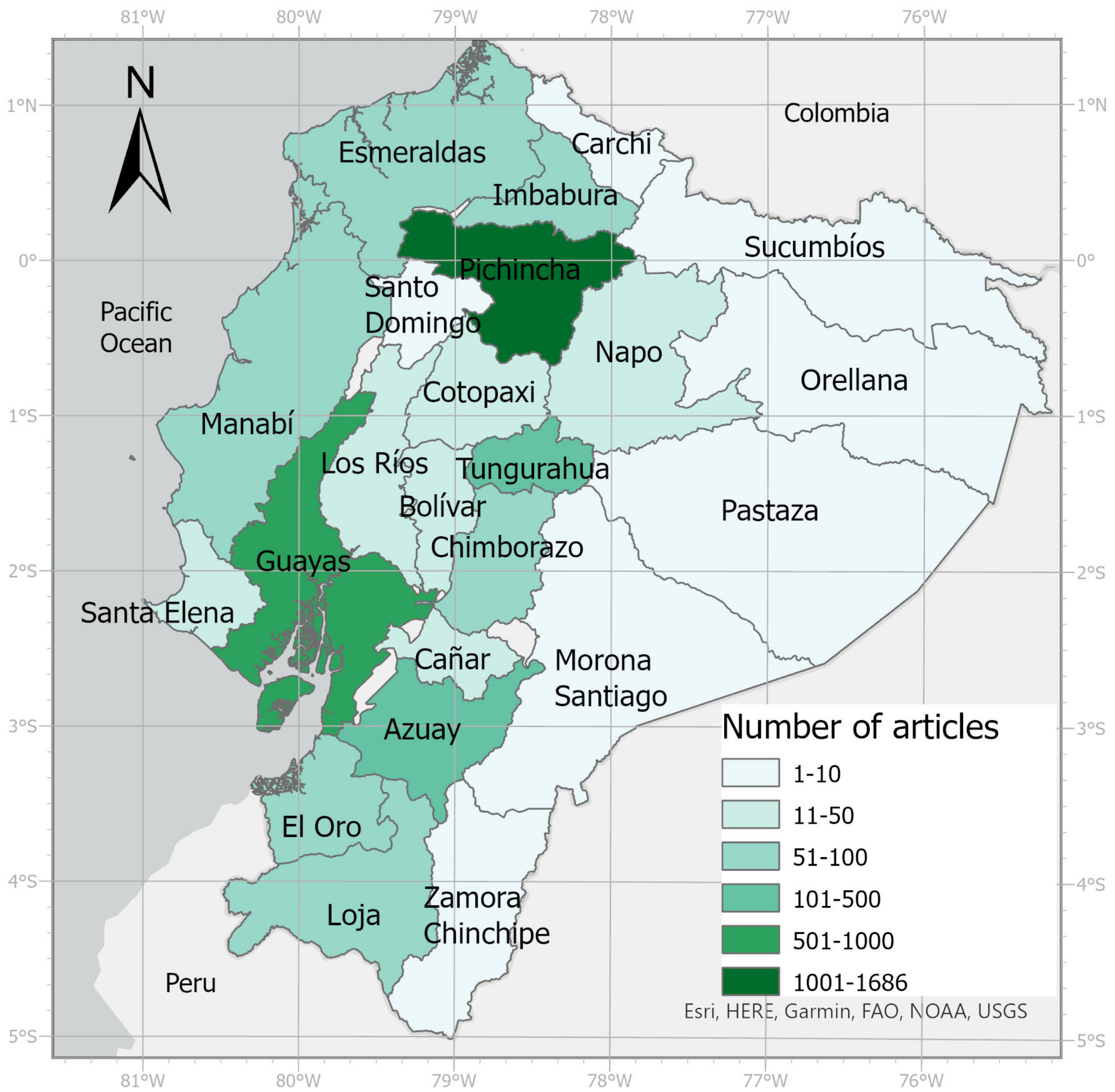

Figure S5. Number of articles per province (N = 3,225)

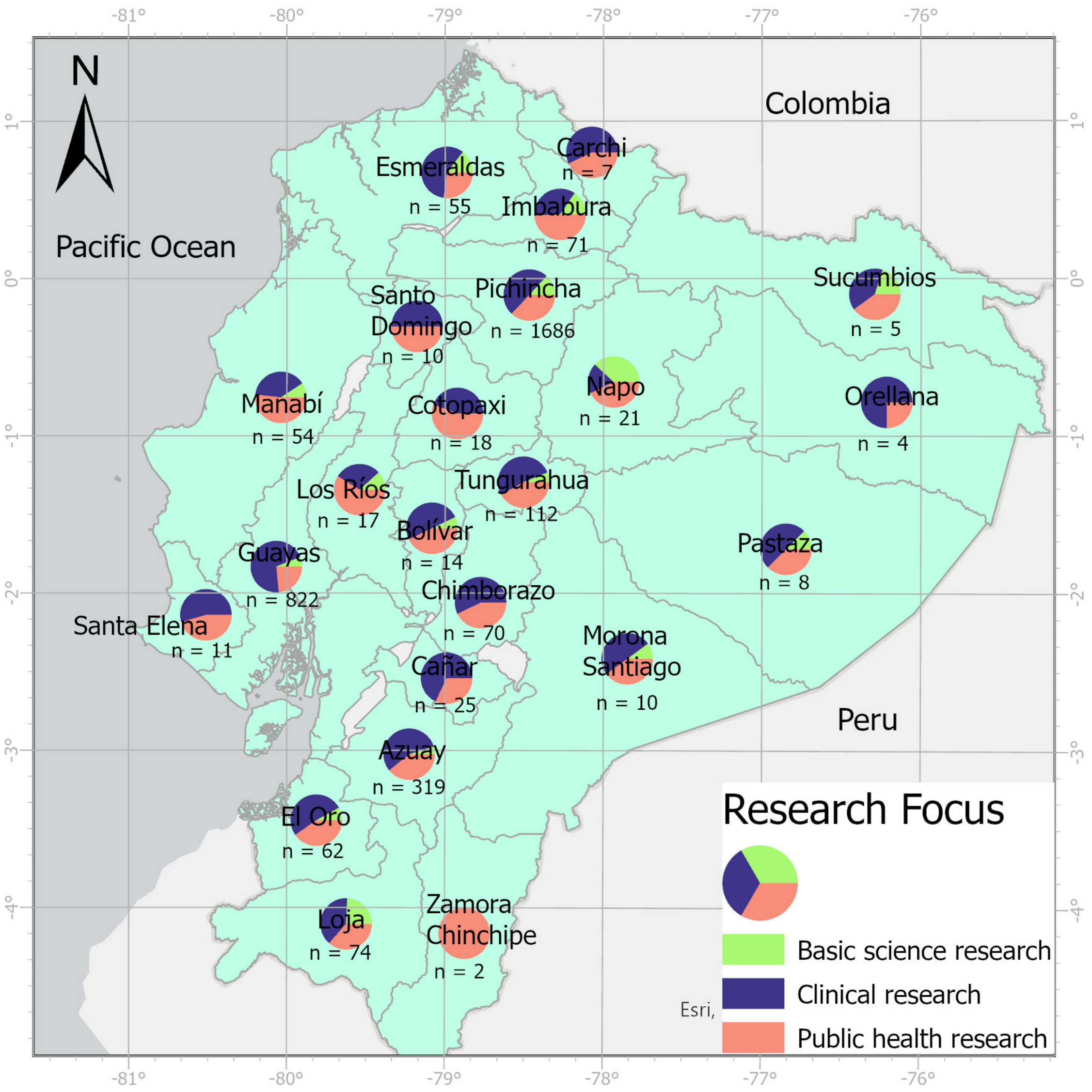

Figure S6. Research focus per province (N = 3,225)
